# Targeting the miR‐96‐5p/Cathepsin B Pathway to Alleviate Neuron‐Derived Neuroinflammation in Alzheimer's Disease

**DOI:** 10.1002/mco2.70368

**Published:** 2025-09-06

**Authors:** Kai Zheng, He‐Zhou Huang, Dan Liu, Nadezda Brazhe, Jiajie Chen, Ling‐Qiang Zhu

**Affiliations:** ^1^ Department of Geriatrics Tongji Hospital Tongji Medical College Huazhong University of Science and Technology Wuhan China; ^2^ Department of Pathophysiology School of Basic Medicine Tongji Medical College Huazhong University of Science and Technology Wuhan China; ^3^ Department of Biophysics Faculty of Biology Moscow State University Moscow Russia

**Keywords:** Alzheimer's disease, cathepsin B, miR‐96, neuroinflammation, potential serum biomarker

## Abstract

Alzheimer's disease (AD) is one of the leading causes of dementia in the elderly, and no effective treatment is currently available. Cathepsin B (CTSB) is involved in key pathological processes of AD, but the underlying mechanisms and its relevance to AD diagnosis and treatment remain unclear. In the present study, we found that CTSB expression was abnormally elevated in the hippocampus of 3×Tg mice and was regulated by miR‐96‐5p. Abnormalities in the miR‐96‐5p/CTSB signaling pathway were detected in the serum of both mild cognitive impairment and AD patients, and the combination of serum miR‐96‐5p and CTSB demonstrated strong diagnostic efficacy for cognitive impairment (AUC = 0.7536). Abnormalities in the miR‐96‐5p/CTSB signaling pathway in AD may be associated with Aβ pathology, and neuronal CTSB can be released extracellularly to reactivate adjacent astrocytes. Ultimately, the reconstitution of the miR‐96‐5p/CTSB signaling pathway effectively rescued astrocyte reactivity and memory impairment in AD. Our findings suggest that the neuron‐derived inflammatory mediator CTSB reactivates adjacent astrocytes and mediates memory impairment in early AD. The combination of serum miR‐96‐5p and CTSB represents potential serum biomarkers for cognitive impairment, and targeting the neuronal miR‐96‐5p/CTSB pathway may serve as a promising therapeutic strategy for AD.

## Graphical abstract

In the AD brain, the Aβ pathology‐dependent miR‐96‐5p/CTSB pathway is dysregulated in neurons, wherein abnormally elevated levels of CTSB are released extracellularly to activate adjacent astrocytes, thereby inducing memory deficits. Furthermore, the miR‐96‐5p/CTSB pathway is regulated by the upstream transcription factor NeuroD2. In the serum of AD patients, miR‐96‐5p expression was decreased, whereas CTSB levels were abnormally elevated, which is consistent with the alterations observed in the brain.

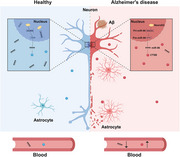

## 1 Introduction

Alzheimer's disease (AD) is a leading cause of dementia in the elderly, currently affecting approximately 50 million people worldwide, with the number of cases continuing to rise [1, 2]. As the disease progresses, people with AD experience cognitive decline and increased healthcare and caregiving costs, placing a significant burden on society. Unfortunately, no clinically effective early diagnostic or therapeutic approaches are currently available. Although the detailed mechanisms of cognitive impairment remain unclear, the deposition of Aβ plaques and tau protein phosphorylation are widely accepted hypotheses [3, 4]. Additionally, neuroinflammation also plays a critical role in AD pathogenesis. In the brains of AD patients, chronic neuroinflammation induced by various pathogenic factors triggers glial cell reactivation, leading to the secretion of pro‐inflammatory and anti‐inflammatory factors, exacerbating neuronal damage [5]. Therefore, elucidating the mechanisms by which pathogenic factor‐induced neuroinflammation contributes to cognitive decline and investigating peripheral alterations in related molecules may facilitate the early diagnosis and treatment of AD.

Recent research has highlighted the significant role of the cathepsin family in neural regulation and neurogenesis across various neurological disorders. Humans possess a total of 15 cathepsins [6], which are widely found in the brain and are essential for various neurological functions, including responses to nerve injury, memory, and cognition [7, 8, 9]. Notably, the expression of cathepsin B (CTSB) is significantly elevated in the brains of various AD model mice [10, 11], where it participates in processes such as neuronal apoptosis and neuroinflammation. CTSB is one of the most abundant cathepsins in neurons, typically localized within intracellular compartments such as endosomes and lysosomes. However, in the context of AD pathology, it can also be released into the extracellular space, contributing to the production of toxic β‐amyloid peptides, thereby damaging neurons [12, 13]. Furthermore, some studies suggest that CTSB drives memory impairment in AD by activating neuroinflammatory responses in microglial cells [14]. In contrast, CTSB knockdown in AβPPLon model mice reduced the expression of toll‐like receptor 2 and IL‐1β in microglia, leading to attenuated inflammation and cognitive improvement [15, 16]. This implies that the abnormal expression of CTSB in AD plays a crucial role in memory impairment, and this role may be associated with interactions between neurons and glial cells. Importantly, CTSB accumulates alongside amyloid plaques in AD brains [17]. By utilizing extracellular vesicle proteomics analysis, a recent study discovered elevated levels of CTSB in the cerebrospinal fluid and plasma extracellular vesicles of AD patients with amyloid progression [18]. This implies that CTSB may serve as a promising candidate biomarker for AD.

MicroRNAs (miRNAs) are short noncoding RNAs that primarily function at the posttranscriptional level. Growing evidence indicates that miRNAs play crucial roles in the pathogenesis of AD. The expression of miR‐9 and miR‐29a is significantly reduced in the AD brain, while the expression of their common target gene β‐secretase 1 is abnormally increased, leading to the elevation of Aβ, which ultimately exacerbates the pathology of AD [19]. Downregulated miR‐135a‐5p targets downstream pathways mediating memory and synaptic damage in the hippocampus of AD model mice [20]. Additionally, numerous miRNAs, including miR‐30b‐5p, miR‐22‐3p, and miR‐29a‐3p, are dysregulated in the peripheral blood of AD patients. These miRNAs are not only involved in the pathological processes of AD but also have potential as diagnostic biomarkers [21, 22]. However, it remains unclear whether CTSB expression is regulated by miRNAs during AD progression, how this signaling pathway functions mechanistically, and whether it contributes to AD diagnosis and therapy.

In this study, we examined the mechanisms underlying the abnormal expression of CTSB during AD progression and explored how the miR‐96‐5p/CTSB signaling pathway contributes to cognition impairment, as well as its potential as a target for early diagnosis and therapeutic intervention in AD.

## 2 Results

### 2.1 Deregulation of the miR‐96‐5p/CTSB Signaling Pathway in the Hippocampus of 3×Tg Mice

**FIGURE 1:**
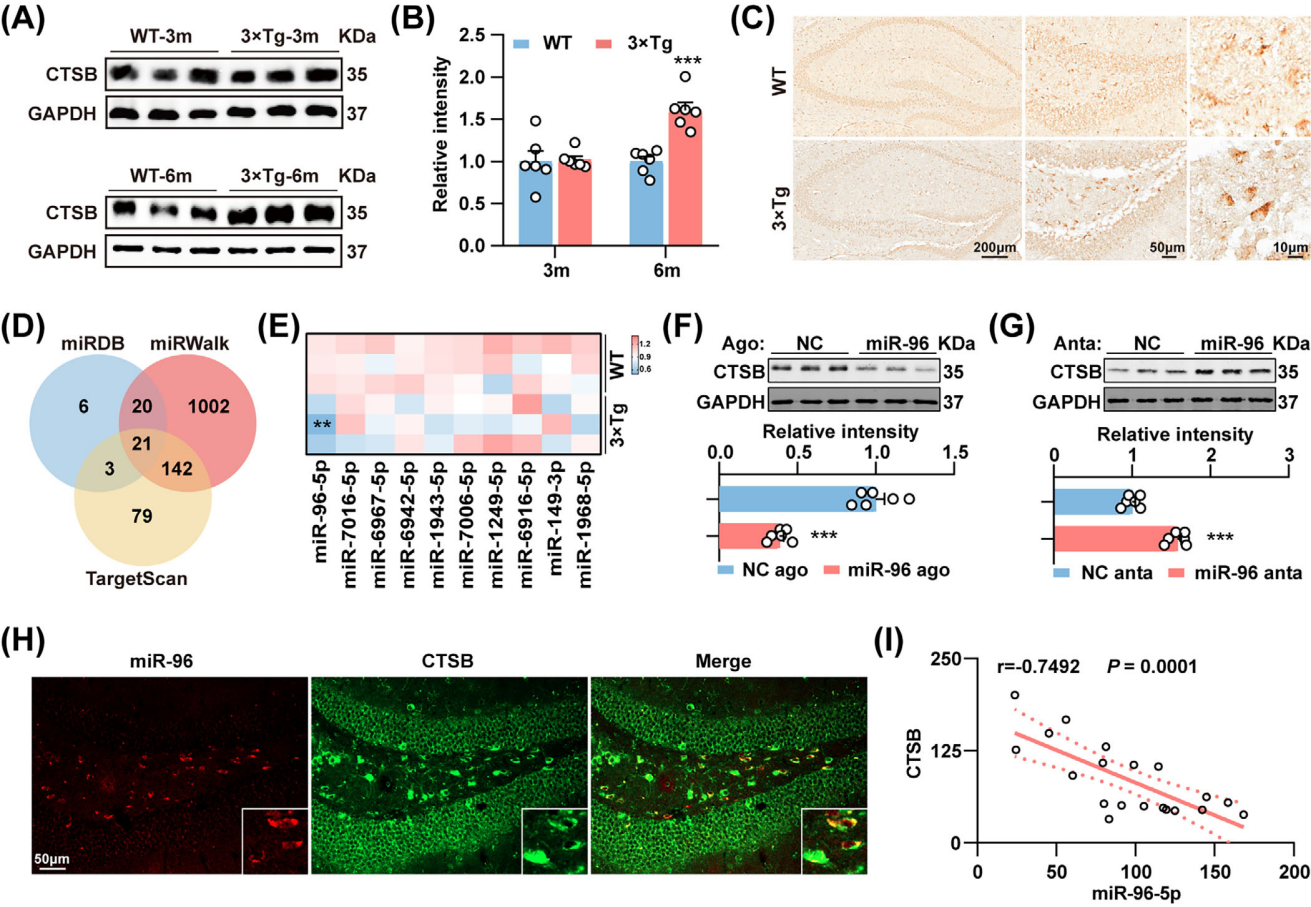
Deregulation of the miR‐96‐5p/CTSB signaling pathway in the hippocampus of 3×Tg mice. (A, B) Immunoblot analysis (A) and quantitative analysis (B) of CTSB and glyceraldehyde phosphate dehydrogenase (GAPDH) in the hippocampus of 3‐month‐old and 6‐month‐old WT mice and 3×Tg mice. *N* = 6. (C) Immunohistochemical staining of hippocampal CTSB was conducted in 6‐month‐old WT mice and 3×Tg mice, with cells in the DG region being magnified. (D) A Venn diagram illustrating the predicted miRNAs that target *Ctsb* translation was presented. (E) qPCR for the top ten candidate miRNAs targeting *Ctsb* in the hippocampus of WT mice and 3×Tg mice. *N* = 3. (F, G) Immunoblot analysis of CTSB in N2a cells transfected with miR‐96‐5p agomirs (miR‐96 ago) or scramble control (NC ago) (F), as well as miR‐96‐5p antagomirs (miR‐96 anta) or scramble control (NC anta) (G). *N* = 6. (H, I) Fluorescence analysis of miR‐96‐5p (red) and CTSB (green) in the DG region of 3×Tg mice (H) and correlation analysis (I). Enlarged sites represent neurons exhibiting higher and lower expressions of miR‐96‐5p. *N* = 20 cells from 4 to 6 slices. *p < 0.05, **p < .01.

To explore the potential role of CTSB in AD, the expression of CTSB in the hippocampus and cortex of 3×Tg mice was examined. Western blot analysis revealed significantly elevated expression of CTSB in the hippocampus of 6‐month‐old 3×Tg mice, with no significant change observed at 3 months of age (Figure 1A, B). However, there was no significant difference in CTSB protein levels in the cortex between wild‐type (WT) mice and 3×Tg mice (Figure S1A,B). Immunohistochemistry confirmed the abnormal upregulation of CTSB in the hippocampal dentate gyrus (DG) hilus of 3×Tg mice (Figure 1C; Figure S1C). Subsequently, we attempted to elucidate the potential molecular mechanism underlying the abnormal upregulation of CTSB in AD. Initially, we examined the levels of *Ctsb* mRNA and found no significant difference between the hippocampus of WT and 3×Tg mice (Figure S1D). These data suggest that the elevated expression of CTSB in the hippocampus of 3×Tg mice might result from posttranscriptional regulation, predominantly mediated by miRNA inhibition. Using a series of bioinformatics tools (TargetScan, miRDB, and miRWalk), we identified twenty‐one miRNAs as candidate regulators of *Ctsb* (Figure 1D; Table S1). We selected the top ten miRNAs (miR‐96‐5p, miR‐7016‐5p, miR‐6967‐5p, miR‐6942‐5p, miR‐1943‐5p, miR‐7006‐5p, miR‐1249‐5p, miR‐6916‐5p, miR‐149‐3p, and miR‐1968‐5p) with the highest scores for further expression analysis in the hippocampus of 3×Tg mice. Among them, only miR‐96‐5p expression was significantly decreased in 6‐month‐old 3×Tg mice (Figure 1E; Figure S1E). Notably, miRNAs exert their biological functions primarily by binding their seed regions to the 3′UTR of specific target genes. To validate the posttranscriptional regulation of CTSB by miR‐96‐5p, we constructed WT and mutant CTSB 3'UTRs, inserted them into a dual luciferase reporter vector, respectively, and transfected 293T cells. The results indicated that miR‐96‐5p agomirs significantly inhibited luciferase activity in cells transfected with the WT 3'UTR (Figure S1F,G). Furthermore, transfection of miR‐96‐5p agomirs or antagomirs into N2a cells resulted in the downregulation or upregulation of CTSB (Figure 1F, G), respectively, without affecting its mRNA expression (Figure S1H–K). Importantly, both miR‐96‐5p and CTSB were detected in the hippocampal DG of 3×Tg mice. However, their expression levels showed a negative correlation (Figure 1H, I). These findings strongly suggest that CTSB is a direct target of miR‐96‐5p, indicating that the miR‐96‐5p/CTSB signaling pathway is disrupted in 3×Tg mice. GO enrichment analysis (https://cloud.oebiotech.com/) of miR‐96 potential target genes (Table S2; Figure S1L) showed that the top 10 GO terms were closely related to neurological functions, such as nervous system development, motor neuron migration, and neural tube closure, suggesting that miR‐96 may regulate multiple aspects of neurological functions.

To delve deeper into the depletion of miR‐96‐5p in AD, we analyzed the levels of both primary and precursor transcripts of miR‐96‐5p. Our findings indicated that both pri‐miR‐96 and pre‐miR‐96 were significantly reduced in 3×Tg mice (Figure S2A,B), suggesting that transcriptional inhibition may underlie the loss of miR‐96‐5p in the AD model. miR‐96, miR‐182, and miR‐183 are clustered together and exhibit comparable expression profiles (Figure S2C). Additionally, pri‐miR‐182 and pri‐miR‐183 were decreased in 3×Tg mice (Figure S2D,E). Utilizing online bioinformatics resources including Jaspar, TransmiR, and animal TFDB, we identified seven transcription factors situated within the promoter regions of miR‐96, miR‐182, and miR‐183 (Figure S2F; Table S3). Through the analysis of their expression in the brain (www.uniprot.org, www.biogps.org), we discovered that Myod1 is expressed at very low levels in the brain. Among the other six transcription factors, we observed a significant decrease in NeuroD2 expression, a significant increase in Smad4 expression, and no notable changes in the mRNA levels of Stat5a, Myc, Tcf3, and Jun (Figure S2G). The overexpression of NeuroD2 in cells transfected with the promoter region of miR‐96 resulted in a significant increase in luciferase intensity (Figure S2H). Consistently, chromatin immunoprecipitation (ChIP)‐seq data (GSE67539) [23] confirmed direct binding of NeuroD2 to the pri‐miR‐96 promoter (Figure S2I). The protein expression of NeuroD2 was significantly reduced in the hippocampus of 3×Tg mice (Figure S2J). In summary, miR‐96‐5p downregulation in AD may be regulated by its upstream transcription factor, NeuroD2.

### 2.2 MiR‐96‐5p Combined with CTSB as Potential Serum Biomarker for AD Diagnosis

**FIGURE 2:**
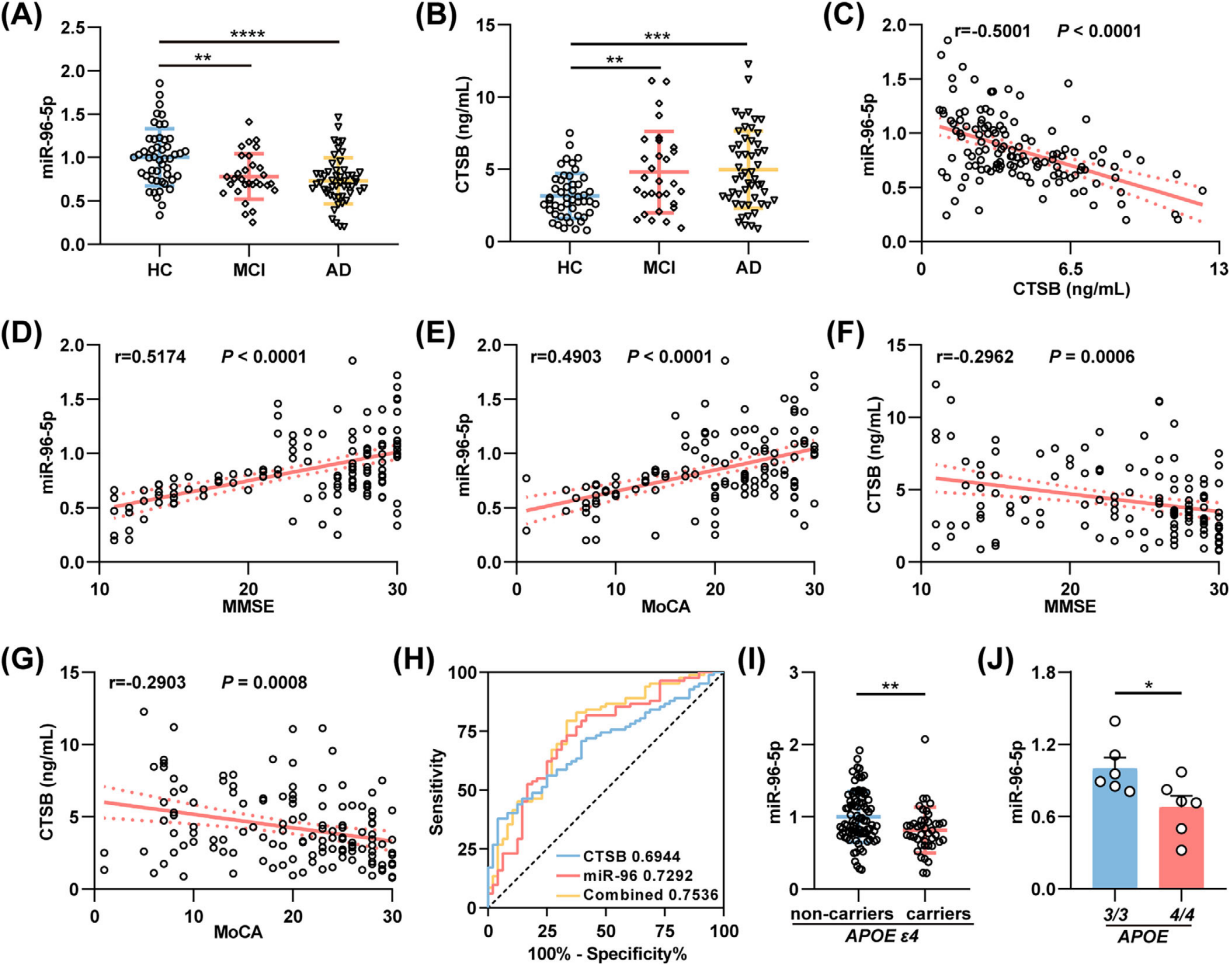
MiR‐96‐5p combined with CTSB as a potential serum biomarker for AD diagnosis. (A) qPCR for the relative expression levels of serum miR‐96‐5p in HC (*N* = 48), MCI (*N* = 30), and AD (*N* = 52) patients. (B) ELISA for the expression of serum CTSB in HC, MCI, and AD patients. (C) A correlation analysis for the relationship between serum miR‐96‐5p and CTSB across all participants. (D, E) Correlation analysis for the relationship between serum miR‐96‐5p and MMSE score (D) as well as MoCA score (E). (F, G) Correlation analysis for the relationship between serum CTSB and MMSE score (F) as well as MoCA score (G). (H) ROC curve analysis for cognitive impairment by serum miR‐96‐5p, CTSB, and the combination of miR‐96‐5p with CTSB across all participants. (I) qPCR for the relative expression of serum miR‐96‐5p in *APOE ε4* noncarriers and carriers. (J) qPCR for the relative expression of serum miR‐96‐5p in *APOE3/3* mice and *APOE4/4* mice. *N* = 6. *p < 0.05, **p < 0.01.

Despite the identification of aberrant miR‐96‐5p/CTSB pathway expression in the hippocampus of 3×Tg mice, it remains unclear whether these alterations are detectable in serum and could serve as potential diagnostic biomarkers. Our findings revealed that serum miR‐96‐5p levels were significantly lower and serum CTSB levels significantly higher in 3×Tg mice compared with WT mice (Figure S3A,B). Additionally, a significant negative correlation was observed between miR‐96‐5p and CTSB levels (Figure S3C). To further elucidate the alterations in miR‐96‐5p/CTSB levels in the serum of AD patients, we collected serum samples from 48 healthy controls (HC), 30 mild cognitive impairment (MCI) patients, and 52 patients with mild to moderate AD (Table 1). Serum miR‐96‐5p levels were significantly lower in both MCI and AD patients compared with HC (Figure 2A), while CTSB levels were significantly higher (Figure 2B). In line with the animal studies, the levels of miR‐96‐5p were negatively correlated with CTSB in these samples (Figure 2C). Furthermore, we analyzed the correlation between serum miR‐96‐5p and CTSB levels with cognitive scores and observed that the correlation coefficients of miR‐96‐5p with MMSE and MoCA scores were 0.5174 and 0.4903, respectively (Figure 2D, E), whereas the correlation coefficients of CTSB with MMSE and MoCA scores were −0.2962 and −0.2903, respectively (Figure 2F,G). Given that MCI and AD represent distinct stages of cognitive decline, the two groups were combined into a single cognitive impairment (CI) group. The receiver operating characteristic (ROC) curve analysis demonstrated that the area under the curve (AUC) for diagnosing CI was 0.7292 for miR‐96‐5p and 0.6944 for CTSB, while the AUC for the combined miR‐96‐5p and CTSB was 0.7536 (Figure 2H). When comparing HC and AD patients separately, the AUCs for miR‐96‐5p, CTSB, and their combination in diagnosing AD were 0.7468, 0.7051, and 0.7748, respectively (Figure S3D). Therefore, the combined serum levels of miR‐96‐5p and CTSB may serve as a potential biomarker for AD diagnosis.

**TABLE 1:** Characteristics of participants and neuropsychological data.

	HC	MCI	AD
Number of patients	48	30	52
Age (years)	70.13 ± 4.68	70.60 ± 3.40	71.75 ± 5.30
Female (%)	27(56.3)	15(50)	28(53.8)
BMI	23.34 ± 2.27	23.60 ± 2.19	22.57 ± 2.34
Education (years)	10.25 ± 2.18	9.63 ± 2.32	8.27 ± 2.57
Smoking (%)	22.9	30.0	30.8
Drinking (%)	6.3	20.0	26.9
Hypertension (%)	43.8	53.3	65.4
CHD (%)	20.8	26.7	30.8
Diabetes (%)	22.9	33.3	36.5
Hyperlipidemia (%)	16.7	13.3	21.2
*ApoE ε4* carriers (%)	20.8	30.0	44.2
MMSE	29.00 ± 0.89	26.20 ± 1.05	17.04 ± 4.22
MoCA	26.92 ± 2.44	22.53 ± 2.29	11.54 ± 4.89

*Note*: Continuity variables were shown as mean ± SD, and categorical variables were shown as proportions.

Abbreviations: BMI, body mass index; CHD, coronary heart disease; MoCA, Montreal Cognitive Assessment; MMSE, Mini‐Mental State Examination.

When participants were grouped according to different AD risk factors, a significant reduction in serum miR‐96‐5p levels was observed only in *APOE ε4* allele carriers. In contrast, factors such as gender, education, smoking, drinking, hypertension, coronary heart disease, diabetes, and hyperlipidemia had no significant effect on miR‐96‐5p expression (Figure 2I; Figure S3E–L). In addition, miR‐96‐5p expression levels in the serum of *APOE4/4* mice were significantly lower than those in *APOE3/3* mice (Figure 2J). Furthermore, in the hippocampus of *APOE4/4* mice, miR‐96‐5p was slightly downregulated (Figure S4A–C). Similarly, NeuroD2 exhibited a slight decrease in *APOE4/4* mice, although this was not statistically significant (Figure S4D). Overall, these findings strongly suggest that dysregulation of the miR‐96‐5p/CTSB signaling pathway in AD is associated with the *APOE ε4* allele.

### 2.3 Aβ but Not Tau Pathology Triggers the Aberrant Neuronal miR‐96‐5p/CTSB Signal in AD

**FIGURE 3:**
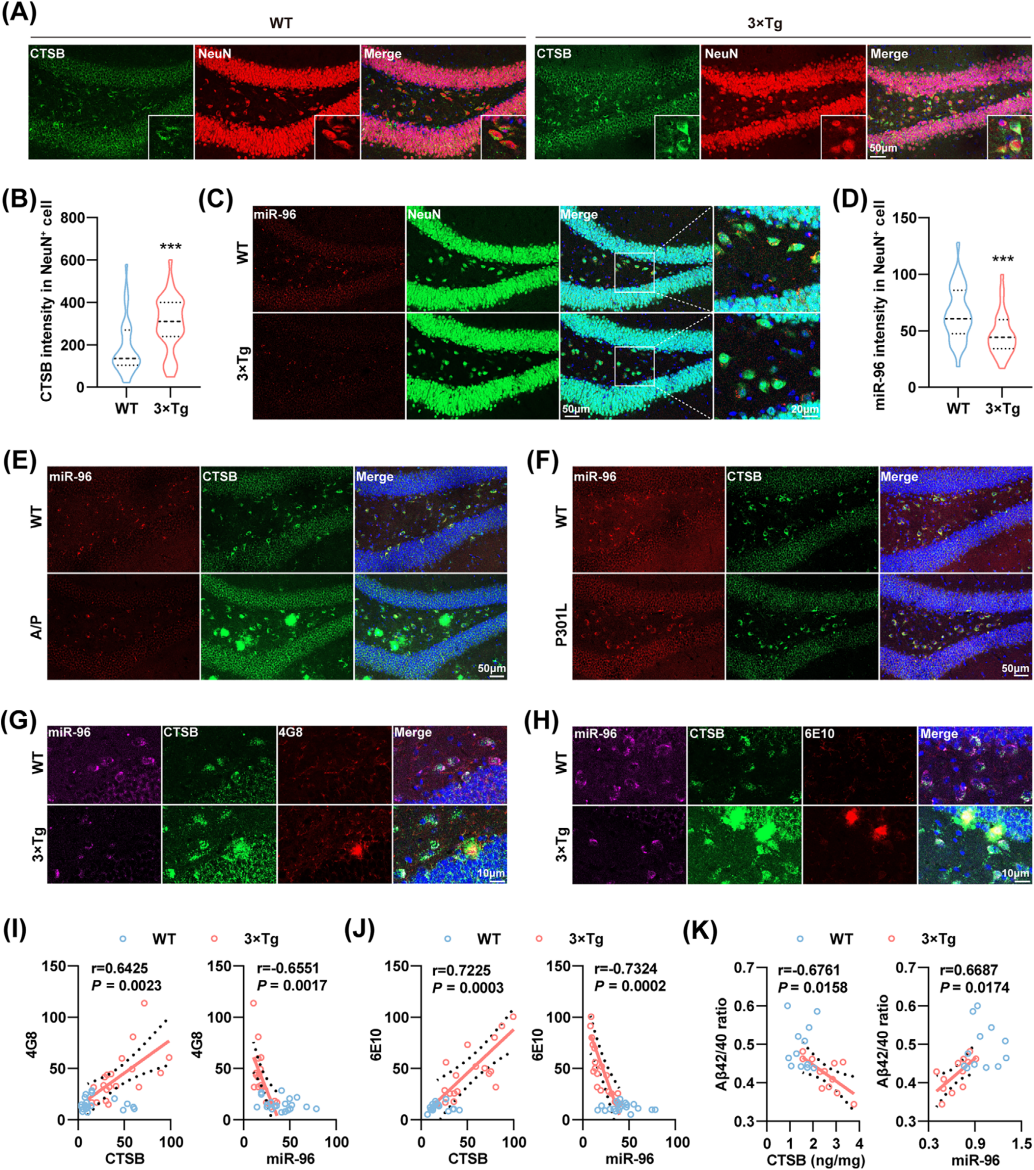
Aβ pathology triggers the aberrant neuronal miR‐96‐5p/CTSB signaling in AD. (A, B) Immunofluorescence staining of CTSB (green) and NeuN (red) in the DG region of WT mice and 3×Tg mice (A), along with fluorescence intensity statistics for CTSB in NeuN^+^ cells (B). *N* = 35 neurons from 5 to 6 mice. (C, D) Immunofluorescence staining of miR‐96‐5p (red) and NeuN (green) in the DG region of WT mice and 3×Tg mice (C), along with fluorescence intensity statistics for miR‐96‐5p in NeuN^+^ cells (D). *N* = 35 neurons from 5 to 6 mice. (E) Representative images of miR‐96‐5p (red) and CTSB (green) in the DG region of WT mice and APP/PS1 (A/P) mice. (F) Representative images of miR‐96‐5p (red) and CTSB (green) in the DG region of WT mice and P301L mice. (G, H) Representative images of miR‐96‐5p (purple), CTSB (green), and 4G8 (red) (G) or 6E10 (red) (H) in the DG region of WT mice and 3×Tg mice. (I, J) Correlation analyses of fluorescence intensities of CTSB, miR‐96, and 4G8 (I) as well as 6E10 (J) in the DG region of hippocampus of WT and 3×Tg mice. *N* = 20 cells from 4 to 6 mice. (K) Correlation analysis of the relationship between CTSB, miR‐96, and the Aβ42/40 ratio in the hippocampus of WT mice and 3×Tg mice. *N* = 12. *p < 0.05, **p < 0.01.

Immunofluorescence staining showed a pronounced increase in CTSB levels in the DG region of the hippocampus in 3×Tg mice (Figure 3A; Figure S5A–C). Furthermore, CTSB expression was examined across different cell types in the DG, revealing elevated levels in both neurons and microglia in 3×Tg mice, with a predominant increase in neurons. In contrast, CTSB expression was nearly undetectable in astrocytes and oligodendrocytes (Figure 3A,B; Figure S5D–F). Similarly, miR‐96‐5p was predominantly localized in neurons and was nearly undetectable in other cell types (Figure 3C,D; Figure S5G–I). We then aimed to explore the relationship of the dysregulated neuronal miR‐96‐5p/CTSB signal with the AD pathological hallmarks. In APP/PS1 mice, CTSB protein levels were significantly increased, while miR‐96‐5p levels were markedly decreased in the DG region (Figure 3E; Figure S6A–D). However, in P301L tau mice, in which Aβ pathology is absent, no significant change was found in CTSB levels (Figure 3F; Figure S6E–H).

Importantly, in the DG region of 3×Tg mice, Aβ plaque fluorescence intensity showed a negative correlation with miR‐96‐5p fluorescence and a positive correlation with CTSB fluorescence (Figure 3G–J). The Aβ42/40 ratio in the mouse hippocampus was measured by ELISA and was significantly reduced in 3×Tg mice (Figure S6I). CTSB levels were negatively correlated with the Aβ42/40 ratio, whereas miR‐96‐5p expression showed a positive correlation (Figure 3K). These findings suggest that Aβ pathology may play a critical role in triggering dysregulation of the neuronal miR‐96‐5p/CTSB signaling pathway.

### 2.4 Aberrant Neuronal miR‐96‐5p/CTSB Signal Reactivates Astrocyte in AD

**FIGURE 4:**
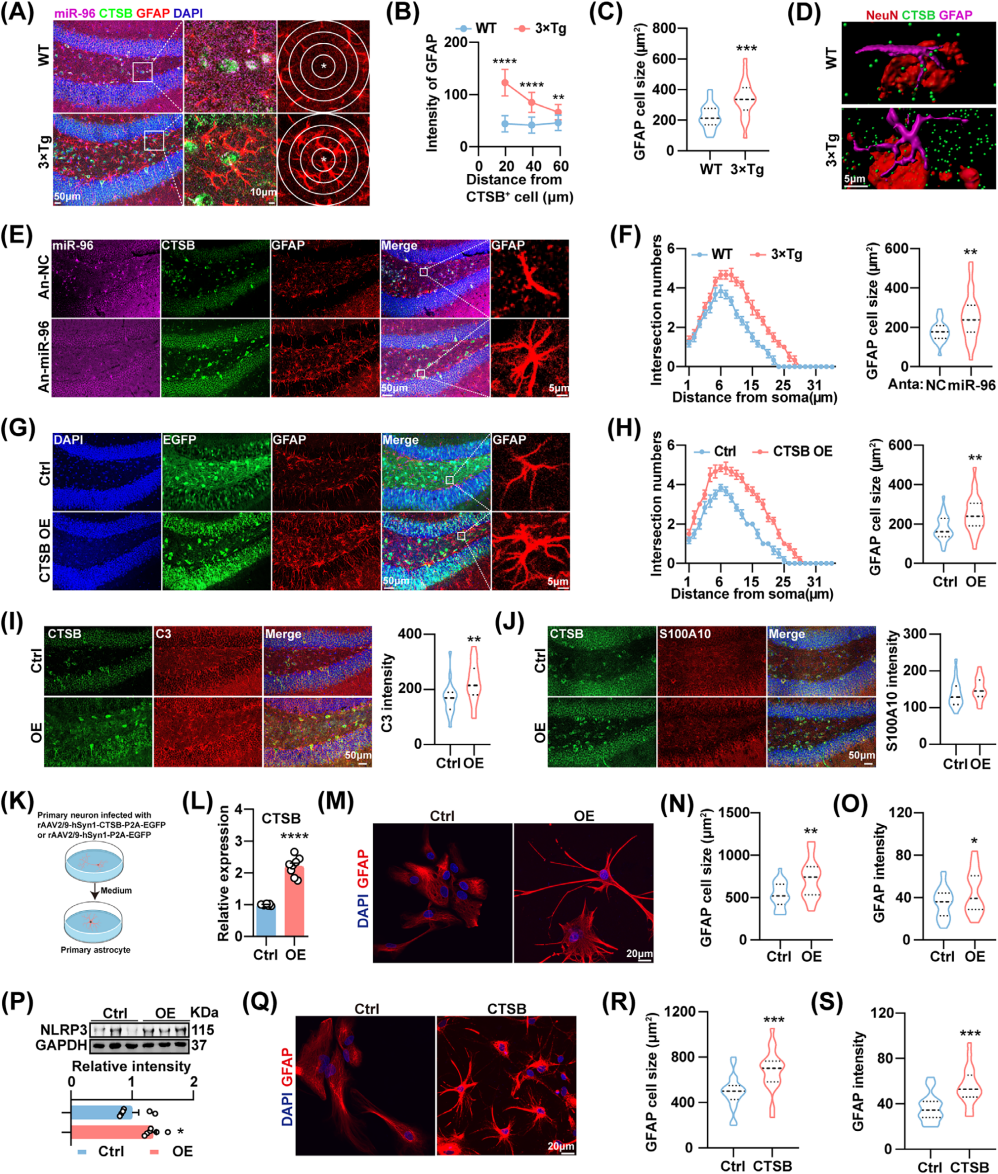
Aberrant neuronal miR‐96‐5p/CTSB signal reactivates astrocyte in AD. (A) Left: Representative images of miR‐96‐5p (purple), CTSB (green), and GFAP (red) in the DG region of WT mice and 3×Tg mice. Middle and right: Immunofluorescence images focused on the center of CTSB^+^ cells (asterisks). (B, C) Fluorescence intensity statistics for GFAP based on the distance from the center of CTSB^+^ cell (B) and statistics on astrocyte cell size (C). *N* = 20 from 4 to 5 mice. (D) Three‐dimensional reconstructed images of NeuN (red), CTSB (green), and GFAP (purple) in the DG region of WT mice and 3×Tg mice. (E–H) Immunofluorescence staining of the DG region of 6‐month‐old WT mice injected with miR‐96‐5p antagomirs (Anta‐miR‐96) or a scramble control (An‐NC) (E), and the DG region of 6‐month‐old WT mice specifically overexpressing CTSB in neurons using AAV (G). The cell size and number of process intersections branching from astrocytes were quantified (F, H). *N* = 25 to 28 from 6 mice. (I, J) Representative images of CTSB and C3 (I) and S100A10 (J) in the DG region of 6‐month‐old WT mice that specifically overexpressed CTSB with AAV. *N* = 20 from 4 mice. (K) Illustration of primary astrocytes cultured in a conditioned medium of primary neurons infected with the virus. (L) ELISA for the relative expression of CTSB in the conditioned medium of virus‐infected primary neurons. *N* = 8. (M–O) Immunofluorescence staining of primary astrocytes (M), along with statistics on cell size (N) and fluorescence intensity (O) of GFAP^+^ astrocytes. *N* = 26 astrocytes. (P) Immunoblot analysis and quantitative analysis of primary astrocytes nod‐like receptor protein 3 (NLRP3) and GAPDH. *N* = 6. (Q–S) Immunofluorescence staining of primary astrocytes treated with CTSB recombinant protein (Q), along with statistics on cell size and fluorescence intensity of GFAP^+^ astrocytes (R, S). *N* = 20 astrocytes. *p < 0.05, **p < 0.01.

We then investigated the contribution of the dysregulation of the miR‐96‐5p/CTSB signal to the pathogenesis of AD. We observed an increased presence of GFAP^+^ astrocytes around CTSB^+^ cells, in which miR‐96‐5p signaling was also abnormal in 3×Tg mice (Figure 4A–C). Furthermore, CTSB signaling inside and outside neurons adjacent to reactivated astrocytes was significantly elevated (Figure 4D). These data indicated that neuronal miR‐96‐5p/CTSB signaling may be involved in astrocyte reactivity in 3×Tg mice. Notably, the infusion of miR‐96‐5p antagomirs or the overexpression of CTSB in neurons (Figure S7A–E) effectively induced reactivation of astrocytes in the hippocampus of WT mice (Figure 4E–H). Overexpression of neuronal CTSB promoted A1‐type reactive astrocytes but not A2‐type astrocytes (Figure 4I,J). It is well established that A1 astrocytes are closely associated with neuroinflammation and neurotoxicity. CTSB, as a protease, can activate NLRP3 inflammasome signaling to induce inflammatory responses [24, 25]. Subsequently, we investigated whether the upregulation of neuronal CTSB promotes astrocyte reactivity and inflammation. CTSB concentration in the culture medium (CM) of primary neurons was significantly increased following AAV‐mediated overexpression of CTSB (Figure 4K,L). This was accompanied by reduced dendritic complexity and decreased cell viability in primary neurons (Figure S7F,G). Treatment of primary astrocytes with CM significantly enhanced astrocyte reactivation, decreased phagocytic activity, and increased NLRP3 expression, thereby inducing an inflammatory response (Figure 4M–P; Figure S7H,I). Similarly, treatment of primary microglia with CM also induced mild activation (Figure S7J,K). Likewise, addition of recombinant CTSB to the CM of primary astrocytes also induced astrocyte reactivation (Figure 4Q–S). CTSB expression was elevated in primary neurons derived from APP/PS1 mice and in their corresponding CM (Figure S7L,M). Moreover, the CM induced astrocyte reactivation and a subsequent inflammatory response (Figure S7N–Q). These findings indicate that neuron‐derived CTSB may trigger astrocyte reactivity and inflammation.

### 2.5 Artificially Simulation of the Aberrant Neuronal miR‐96‐5p/CTSB Pathway Reproduces the AD‐Like Behavior Abnormalities

**FIGURE 5:**
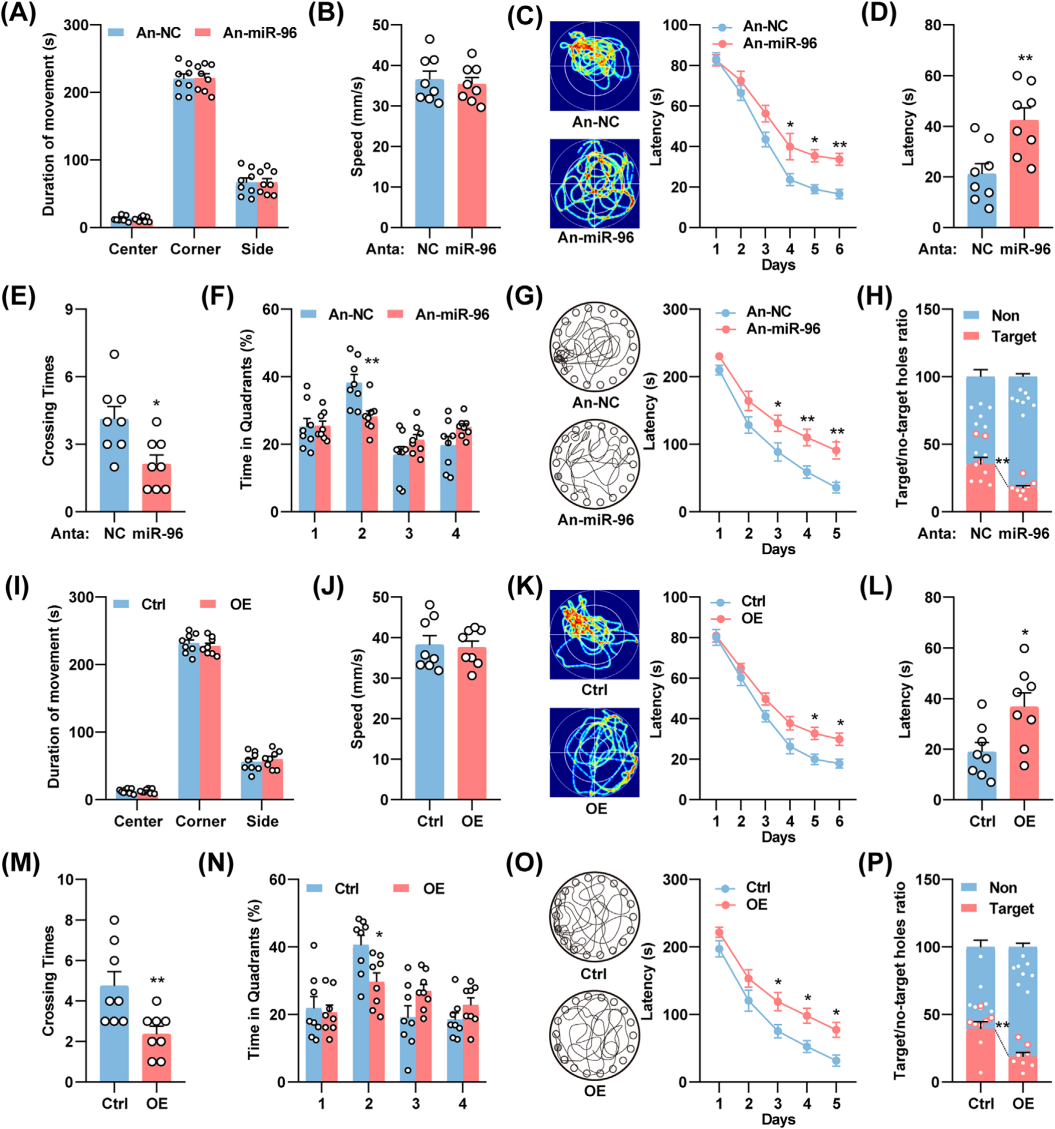
Artificial simulation of the aberrant neuronal miR‐96‐5p/CTSB pathway reproduces the AD‐like behavior abnormalities. (A, B) miR‐96‐5p antagomirs (Anta‐miR‐96) or scramble control (An‐NC) were injected into the DG region of 6‐month‐old WT mice. Residence time (A) and movement speed (B) of the mice in each region of the open field experiment were recorded. *N* = 8. (C–F) Representative traces on day 8 (left) and latency to reach the hidden platform on days 1–6 (right) (C), latency to reach the platform area on day 8 (D), number of crossings in the platform area (E), and percentage of time spent in each quadrant (F) in the Morris water maze experiment. *N* = 8. (G, H) Representative movement trajectories of mice on day 7 (left) and latency to enter the target hole on days 1–5 (right) in the Barnes maze experiment (G), along with accuracy in locating the target hole on day 7 (H). *N* = 8. (I, J) Adeno‐associated virus (AAV) with neuron‐specific overexpression of CTSB (OE) or a negative control (Ctrl) was injected into the DG region of 5‐month‐old WT mice. Residence time (I) and movement speed (J) of the mice in each region of the open field experiment were recorded. *N* = 8. (K–N) Representative traces on day 8 (left) and latency to reach the hidden platform on days 1–6 (right) (K), latency to reach the platform area (L), number of crossings in the platform area (M), and percentage of time spent in each quadrant (N) for mice of the Morris water maze experiment. *N* = 8. (O, P) Representative movement trajectories of mice on day 7 (O) and latency to enter the target hole on days 1–5 (P), along with accuracy in locating the target hole on day 7 (P). *N* = 8. *p < 0.05, **p < 0.01.

To further clarify the essential role of the neuronal miR‐96‐5p/CTSB pathway in the pathogenesis of AD, we conducted behavioral tests on WT mice that were injected with miR‐96‐5p antagomirs or a virus overexpressing CTSB. The open‐field test confirmed that the inhibition of miR‐96‐5p did not influence the emotional state or motor abilities of the mice (Figure 5A,B). However, miR‐96‐5p inhibition significantly impaired learning and memory, as evidenced by increased latency to locate the platform in the Morris water maze, extended time to locate the original platform, fewer platform crossings, and reduced time spent in the target quadrant (Figure 5C–F). In the Barnes maze test, mice exhibited prolonged latency in locating the target hole and made more errors during the search phase (Figure 5G,H). Similarly, overexpression of CTSB in hippocampal neurons did not affect locomotor performance (Figure 5I,J); however, it resulted in increased latency to locate the original platform, fewer platform crossings, and reduced time spent in the target quadrant in the Morris water maze, as well as prolonged latency and decreased exploration of the target hole in the Barnes maze (Figure 5K–P). These results suggest that artificial simulation of the neuronal miR‐96‐5p/CTSB pathway induces AD‐like behavioral deficits in WT mice.

### 2.6 Restoration of the miR‐96‐5p/CTSB Pathway Rescues Memory Impairment and Aberrant Astrocyte Reactivity in 3×Tg Mice

**FIGURE 6:**
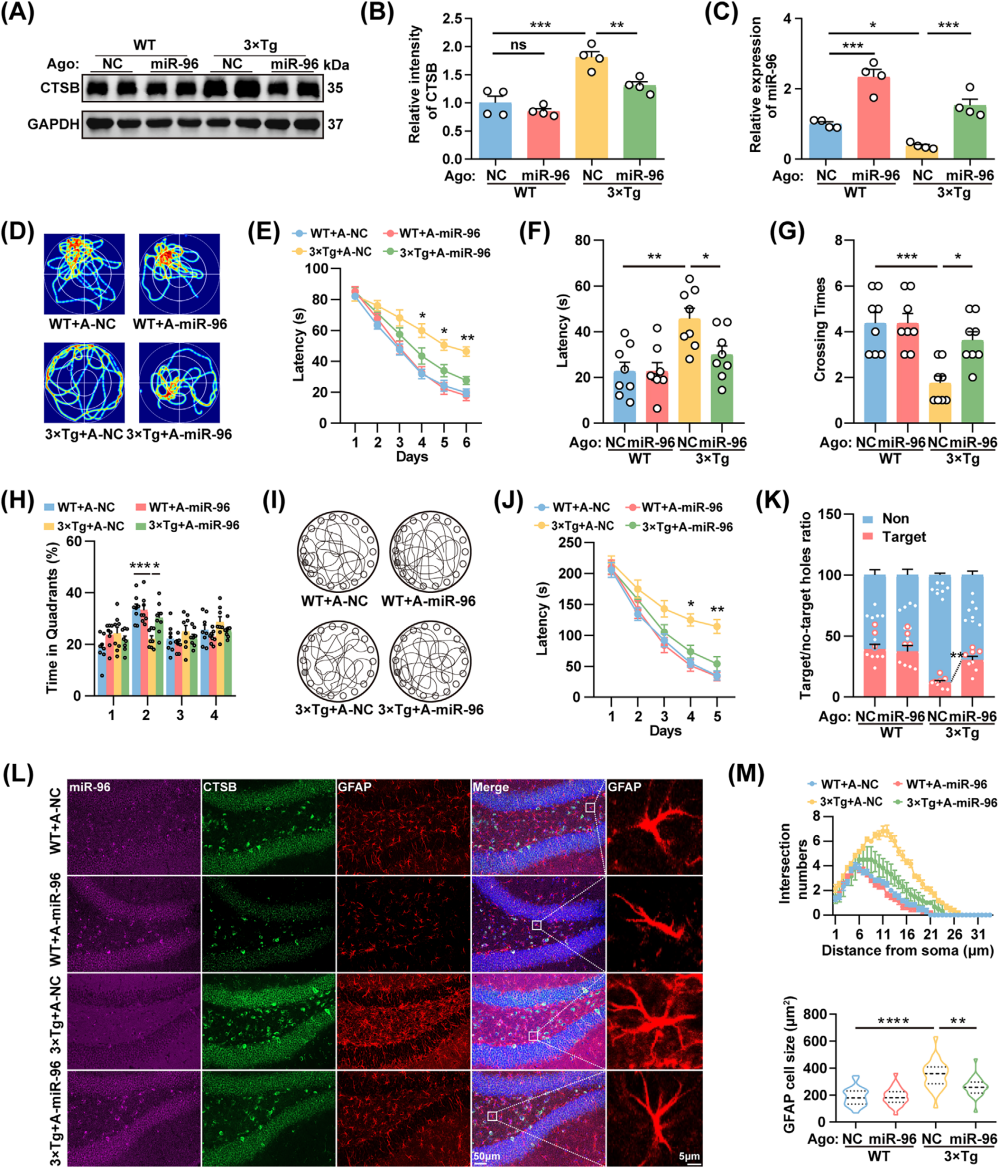
Restoration of miR‐96‐5p signaling pathway rescues memory defects and aberrant astrocyte reactivity in 3×Tg mice. miR‐96‐5p agomirs (A‐miR‐96) or a scramble control (A‐NC) were injected into the DG region of 6‐month‐old WT or 3×Tg mice. (A, B) Immunoblot analysis (A) and quantitative analysis (B) of CTSB in the hippocampus of mice. *N* = 4. (C) qPCR for relative expression of miR‐96‐5p in the hippocampus of mice. *N* = 4. (D‐H) Representative traces on day 8 (D) and latency to reach the hidden platform on days 1–6 (E), latency to reach the platform area on day 8 (F), number of crossings in the platform area (G), and percentage of time spent in each quadrant (H) in the Morris water maze experiment. *N* = 8. (I–K) Representative movement trajectories of mice on day 7 (I) and latency to enter the target hole on days 1–5 (J), along with accuracy in locating the target hole on day 7 (K) in the Barnes maze experiment. *N* = 8. (L, M) Representative images of the DG region for miR‐96‐5p (purple), CTSB (green), and GFAP (red) (L), and statistics on the number of process intersections and cell size of GFAP^+^ astrocytes (M). *N* = 25 from 6 mice. *p < 0.05, **p < 0.01.

**FIGURE 7:**
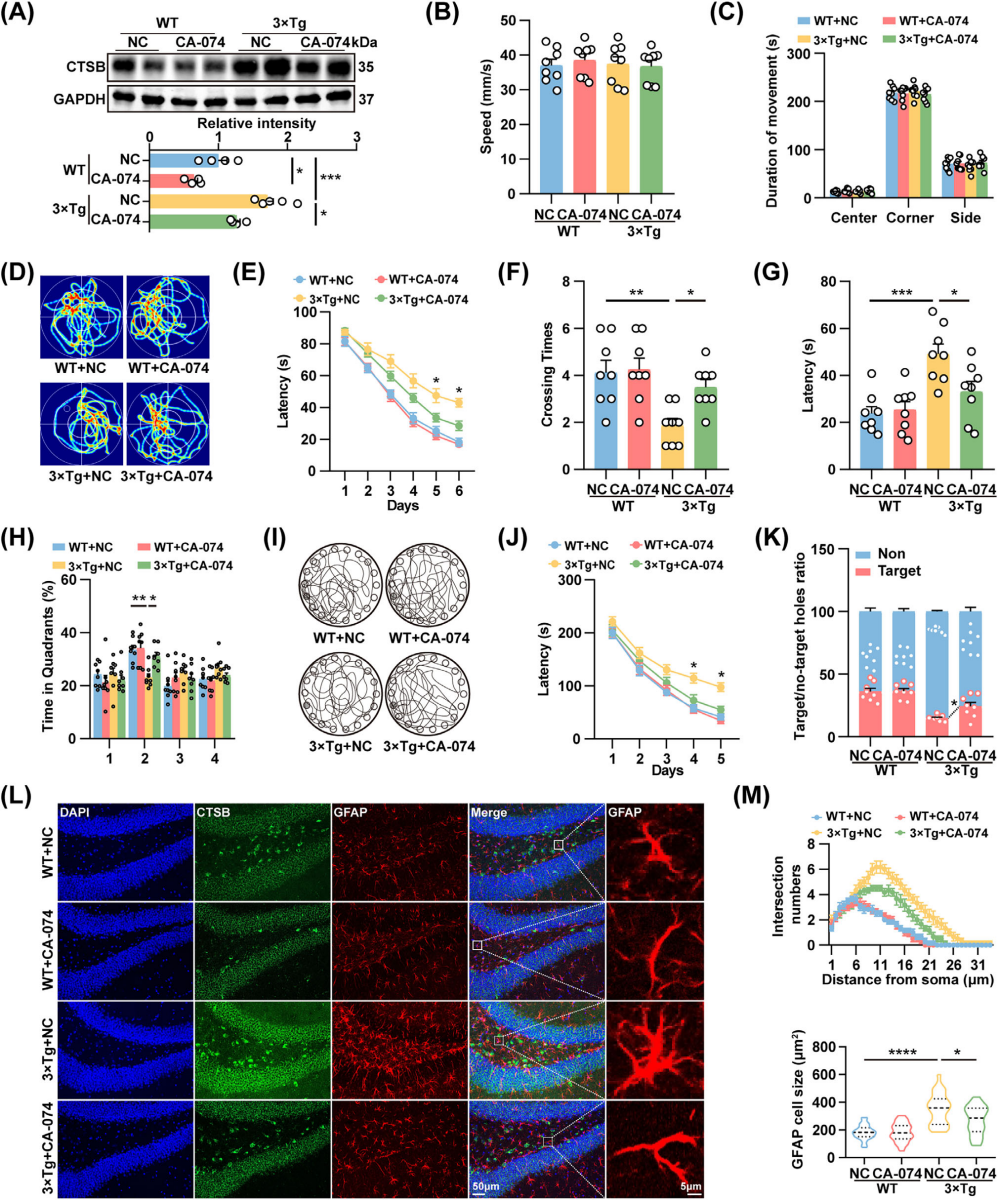
Inhibition of CTSB rescues memory deficits and aberrant astrocyte reactivation in 3×Tg mice. 5.5‐month‐old WT and 3×Tg mice were injected intraperitoneally with the CTSB inhibitor CA‐074 or saline for a duration of 15 days. (A) Immunoblot analysis and quantification of CTSB and GAPDH in the hippocampus of mice. *N* = 4. (B, C) Statistics of residence time (B) and movement speed (C) of mice in each area during the open field experiment. *N* = 8. (D–H) Representative traces on day 8 (D) and latency to reach the hidden platform on days 1–6 (E), latency to reach the platform area on day 8 (F), number of traversals in the platform area (G), and percentage of time spent in each quadrant (H) for mice in the Morris water maze experiment. *N* = 8. (I–K) Representative movement trajectories of mice on day 7 (I) and latency to enter the target hole on days 1–5 (J), and accuracy in locating the target hole on day 7 (K) in the Barnes maze experiment. *N* = 8. (L, M) Representative immunofluorescence staining images of the DG region of the hippocampus for miR‐96‐5p (purple), CTSB (green), and GFAP (red) (L), along with the statistics of process intersections and cell size of GFAP‐positive astrocytes (M). *N* = 25 from 6 mice. *p < 0.05, **p < 0.01.

We then investigated whether correcting the abnormal miR‐96‐5p/CTSB signaling could attenuate astrocyte reactivity and improve learning and memory deficits in AD models. One week after stereotactic injection into the DG region, treatment with miR‐96‐5p agomirs significantly reduced CTSB protein levels in 3×Tg mice, without affecting its mRNA expression (Figure 6A–C). Correction of miR‐96‐5p did not impact the emotional state of the 3×Tg mice (Figure S8A,B); however, it significantly reduced the latency to reach the platform during the training phase and increased the number of platform crossings and time spent in the target quadrant during the Morris water maze (Figure 6D–H). Furthermore, in the Barnes maze task, the latency to find the target hole was reduced, and the proportion of exploration in the target hole was increased (Figure 6I–K). The miR‐96‐5p agomirs also significantly suppressed astrocyte reactivity in 3×Tg mice (Figure 6L,M).To confirm the important role of CTSB in the astrocyte reactivity and memory impairments, CA‐074, a Cathepsin B inhibitor [26], was intraperitoneally injected into 3×Tg mice at a dosage of 10 mg/kg for 15 days to inhibit CTSB expression (Figure 7A). Behavioral assessments revealed that the emotional state of the mice did not change significantly after the inhibition of CTSB (Figure 7B,C). However, the latency of 3×Tg mice to find the platform in the Morris water maze task was significantly reduced, and the frequency of crossing the platform and time spent in the target quadrant were significantly increased (Figure 7D–H). In the Barnes Maze task, the time to find the target hole was shorter, and the proportion of exploration in the target hole was higher (Figure 7I–K). CA‐074 also effectively suppressed astrocyte reactivity in 3×Tg mice (Figure 7L,M). Therefore, the correction of abnormal miR‐96‐5p/CTSB signaling may reduce astrocyte reactivity and alleviate learning and memory deficits in 3×Tg mice.

## 3 Discussion

In this study, we observed increased CTSB protein expression in the hippocampus of 3×Tg and APP/PS1 AD model mice, consistent with previous findings [15]. Subsequently, we examined the cellular distribution of CTSB and found it to be predominantly localized in neurons, with significantly elevated levels in hippocampal neurons of 3×Tg mice. Several studies have reported increased expression of CTSB in microglia during AD progression [27], and our results were consistent with these findings. Given that the majority of CTSB is localized in neurons, the present study focused on the role of its aberrant upregulation in neuronal compartments during AD progression. Neuronal CTSB is typically confined to lysosomes; however, lysosomal leakage during AD progression may lead to its redistribution into the cytoplasm, thereby triggering inflammatory responses and promoting neuronal cell death [28]. Therefore, the localization of CTSB within the cell is crucial for its function. In our study, we observed pronounced astrocyte reactivity adjacent to CTSB^+^ neurons in the DG region of the hippocampus in 3×Tg mice. We also noted a significant increase in both intracellular and extracellular CTSB in neurons near reactivated astrocytes, as well as an increase in CTSB concentration in the CM of primary neurons following specific overexpression of CTSB. Overexpression of CTSB in hippocampal neurons of WT mice also promoted astrocyte reactivity and resulted in AD‐like cognitive impairment. These findings suggest that aberrantly elevated neuronal CTSB in AD may be secreted into the extracellular space, where it reactivates adjacent astrocytes and contributes to cognitive decline.

Aberrant activation of neuroglial cells can mediate neuroinflammation and lead to neurodegenerative disorders [29]. In AD, astrocytes shift from the anti‐inflammatory A2 phenotype to the proinflammatory A1 phenotype, thereby exacerbating pathology through inflammatory factor secretion and impaired Aβ phagocytosis [30]. Although chronic inflammation in AD is commonly attributed to the response to Aβ plaque deposition, neuron‐specific inflammatory responses may precede the formation of Aβ plaques and tau tangles in disease‐susceptible regions of the human brain [31]. Several chemotactic and proinflammatory factors, including CSF‐1, CCL2, CCL3, and IL‐6, can be produced by neurons months before initial plaque deposition [31]. These findings suggest that neuron‐derived proinflammatory factors play a pivotal role in initiating neuroinflammation in AD. CTSB is a proinflammatory factor closely related to inflammatory responses; it promotes inflammation by directly activating the NLRP3 inflammasome and inducing pyroptosis [32, 33]. To assess astrocyte reactivation and inflammation induced by neuronal CTSB, we examined astrocytes exposed to CM from CTSB‐overexpressing neurons. These astrocytes exhibited elevated NLRP3 expression, reduced expression of phagocytosis‐related genes (*Megf10* and *Mertk*), and increased levels of proinflammatory markers (*IL‐1β*, *IL‐6*, and *TNF‐α*). These findings align with CTSB‐induced A1 astrocyte polarization in WT mice, implicating neuronal CTSB in neurodegenerative inflammation. While previous studies have primarily focused on intracellular CTSB–NLRP3 signaling, the mechanism by which soluble CTSB activates the NLRP3 inflammasome remains unclear. We speculate that it may be related to the uptake of aberrantly expressed soluble CTSB by astrocytes through endocytosis [34]. Additionally, primary microglia treated with CM from CTSB‐overexpressing neurons also showed signs of activation. Previous studies have mainly concentrated on the activating and proinflammatory effects of aberrant CTSB expression in microglia [35, 36]. The potential involvement of intercellular soluble CTSB in regulating microglial function in disease states offers new avenues for future investigations. Moreover, CTSB, which is abundantly expressed in neurons, plays a complex role in regulating neuronal function. On one hand, elevated levels of neuronal CTSB can induce neuronal death [37, 38], possibly due to the cleavage of critical proteins necessary for cell survival by aberrantly expressed CTSB [39]. On the other hand, the secretion of lysosomal CTSB can promote neuronal axon growth by degrading chondroitin sulfate [40]. In this study, we employed adeno‐associated viral vectors to overexpress CTSB in primary neurons, which significantly reduced neuronal morphological complexity and cell viability. This effect may be attributed to the rapid and excessive increase in CTSB expression. Collectively, these findings imply that neuronal CTSB plays a crucial role in various brain cell types, promoting adjacent astrocyte reactivation and inducing inflammation that contributes to AD progression.

We further investigated the molecular mechanisms underlying the upregulation of CTSB in AD and found that only the protein expression of CTSB was increased in the hippocampus of AD mice. In a subsequent study, we discovered that the upregulation of CTSB was due to the downregulation of miR‐96‐5p in the AD hippocampus. In the nervous system, miR‐96 is widely distributed and essential for maintaining the functional maturation of the auditory system [41]. The circadian rhythm of miR‐96‐5p may regulate the level of neuronal glutathione through EAAC1 [42], potentially playing a neuroprotective role. Here, we report a reduction in miR‐96‐5p levels in the hippocampus of 3×Tg mice, and this deletion of miR‐96‐5p upregulates CTSB. Furthermore, miR‐96‐5p/CTSB was only abnormal in APP/PS1 mice, with miR‐96‐5p levels negatively correlated with Aβ, and CTSB levels positively correlated with Aβ. This finding was also preliminarily validated in the serum of AD patients, indicating that Aβ pathology triggers abnormal neuronal miR‐96‐5p/CTSB signaling in AD. Notably, in the DG region of APP/PS1 mice, immunofluorescence staining revealed clear co‐localization of CTSB with Aβ plaques. Previous studies have identified CTSB as a candidate β‐secretase that cleaves amyloid precursor protein to produce various forms of Aβ, including the more aggregation‐prone Aβ3‐42 [15, 43]. A recent study reported that increased CTSB CPR‐6 activity in aging nematode *C. elegans* promotes Aβ proteotoxicity, and that knockdown of CPR‐6 attenuates Aβ toxicity by decreasing the expression of swsn‐3 and increasing the level of SMK‐1 protein [44]. This is consistent with the results of the present study, suggesting that in AD, Aβ may upregulate neuronal CTSB expression, and the abnormally elevated CTSB may, in turn, promote Aβ production and aggregation, forming a positive feedback loop. miRNAs possess multitarget regulatory properties, with a single miRNA capable of recognizing and binding to multiple mRNA targets simultaneously. GO enrichment analysis of miR‐96‐5p target genes revealed that miR‐96‐5p may be involved in biological processes such as nervous system development, motor neuron migration, and neural tube closure. Among these, molecules related to nervous system development are closely associated with AD pathology. For example, LMTK2 binds to KLC1 to direct axonal transport of p35, and its loss might contribute to AD [45]. However, the current study did not fully elucidate the specific regulatory mechanism of miR‐96‐5p in AD, and further experiments are needed to validate its targeting role and downstream pathways. By examining the levels of primary and precursor transcripts of miR‐96, we found that its transcription is downregulated in AD. Moreover, the reduction in the expression of NeuroD2 may be the cause of the dysregulation of the miR‐96‐5p/CTSB signaling pathway in AD. NeuroD2 is a member of the NeuroD family of basic helix‐loop‐helix transcription factors, which are mainly involved in the regulation of early neuronal differentiation during development and synapse formation after birth [46]. In the hippocampus, NeuroD2 is also involved in regulating neurogenesis and the development of mossy fiber synapses [47, 48], processes that are closely linked to AD progression. Therefore, the NeuroD2/miR‐96‐5p/CTSB pathway may be involved in the pathogenic process of AD.

Despite ongoing challenges in diagnosing AD, the development of in vivo biomarkers has shifted diagnosis from the advanced dementia stage to earlier stages, introducing the potential for pre‐symptomatic detection [49]. miRNAs are increasingly studied in AD as they are not only key regulators of gene expression but also promising candidates for biomarker development [50]. Research has shown that miR‐331‐3p, miR‐9‐5p, autophagic activity, and amyloid plaques can differentiate between early and late AD, leading to more accurate and timely diagnosis [51]. Compared with HC, miR‐146b‐5p and miR‐15b‐5p in the blood consistently show differential expression in AD and are considered promising biomarkers for AD diagnosis [52]. miR‐96‐5p, the focus of this study, has diagnostic potential in a variety of diseases. In PD patients, miR‐96‐5p levels in serum are significantly decreased and could be used as a biomarker for early diagnosis [53]. In contrast, we reported that miR‐96‐5p expression was significantly reduced in the serum of 3×Tg mice and in patients with MCI and AD, suggesting its potential as a biomarker for cognitive impairment. Several studies have enhanced disease diagnosis efficacy by combining miRNAs with target genes [54, 55]. This study also examined CTSB expression levels in AD serum. We found that serum CTSB levels were significantly elevated in 3×Tg mice and in patients with MCI and AD. The combination of serum miR‐96‐5p and CTSB as potential biomarkers for cognitive impairment significantly enhanced diagnostic sensitivity and specificity. The most widely recognized blood biomarkers for AD diagnosis include characteristic pathological proteins such as Aβ42/40, p‐tau181, p‐tau217, p‐tau231, and neurodegeneration markers such as GFAP and NFL [56]. In a small‐sample clinical study, the AUC of plasma Aβ42/40 for distinguishing amyloid PET‐positive subjects was 0.949, indicating extremely high diagnostic accuracy [57]. The diagnostic accuracy of plasma p‐tau217 and the p‐tau217/Aβ1‐42 ratio (AUC 0.94‐0.97) was significantly higher than that of other markers, including p‐tau181 and Aβ1‐42 [58]. In discriminating MCI from AD, serum GFAP achieved an AUC of 0.77, surpassing that of cerebrospinal fluid Aβ42 and p‐tau181 [59]. Despite the promising diagnostic and differential value of these biomarkers in small sample studies, they are limited by several factors, including poor sensitivity, inadequate protein stability, variability in detection methods, lack of a clear standardized range, and difficulty in differentiating them from similar diseases, which currently hampers their clinical promotion [56]. The rapid advancement of hematological diagnosis for AD is being propelled by multi‐indicator co‐diagnosis, the development of rapid diagnostic kits, and large‐sample baseline data; however, it will still take considerable time before widespread clinical application can be achieved. In this study, although serum miR‐96‐5p combined with CTSB showed promising sensitivity and specificity for diagnosing cognitive impairment in a small‐sample population, validation in a larger population and head‐to‐head comparisons with established diagnostic markers are necessary to further clarify its diagnostic potential and clinical value. Additionally, we explored whether the miR‐96‐5p/CTSB pathway could be a therapeutic target for AD. Briefly, hippocampal infusion of miR‐96‐5p agomirs or intraperitoneal injection of CA‐074, a CTSB inhibitor, reduced astrocyte reactivity in the hippocampus and effectively ameliorated memory impairment in 3×Tg mice. These data suggest that the miR‐96‐5p/CTSB signal is involved in the pathogenic process of AD and further highlight the important role of the miRNA regulatory axis in AD, consistent with our previous reports [60, 61].

In summary, our research highlights elevated CTSB protein levels in the hippocampal neurons of 3×Tg AD model mice, an alteration modulated by miR‐96‐5p. The neuronal inflammatory mediator CTSB reactivates adjacent astrocytes and contributes to memory impairment during AD progression. Additionally, our findings suggest that dysregulation of the miR‐96‐5p/CTSB signaling pathway may serve as a potential serological diagnostic marker and therapeutic target for AD.

## 4 Methods

### 4.1 Participants

This study included 48 HC, 30 patients with MCI, and 52 patients with mild‐to‐moderate AD, all aged 65 or older and recruited from Tongji Hospital, Tongji Medical College, Huazhong University of Science and Technology. HC participants had no cognitive complaints and MMSE scores above 26, with exclusions for organic brain lesions or serious physical illnesses. MCI patients met criteria including subjective or objective memory loss, MMSE ≥ 24, CDR = 0.5, and did not fulfill the diagnostic standards for dementia or AD. AD patients met the NINCDS‐ADRDA criteria, had MMSE scores between 10 and 24, and CDR > 0.5. Exclusion criteria for MCI and AD groups included cognitive impairment due to other conditions or a history of major psychiatric or neurological disorders such as Parkinson's disease, epilepsy, depression, or traumatic brain injury.

### 4.2 Mice

In this study, mice were purchased from Jackson Laboratory (Bar Harbor, ME), including C57BL/6J mice (catalog no. 039777), 3×Tg mice (catalog no. 34830), APP/PS1 mice (catalog no. 034832), P301L mice (catalog no. 024854), *APOE4/4* mice (catalog no. 027894), and *APOE3/3* mice (catalog no. 039777). Only male mice were used in this study. All mice were maintained in a controlled environment with a 12 h light/dark cycle, ensuring appropriate temperature and access to sufficient food and water.

### 4.3 Cell Culture

N2a and HEK293T cells were cultured in DMEM high‐glucose medium supplemented with 10% fetal bovine serum (FBS) in an incubator. The transfection reagent utilized in this study was Lipofectamine 3000 (Invitrogen, Carlsbad, CA, USA), and the procedure was carried out in accordance with the provided instructions.

Primary neurons from C57BL/6J or APP/PS1 mice were isolated and cultured according to previously established protocols [62]. Fetal mice hippocampus were extracted on gestational days 16 or 17 from female mice, and blood vessels and meninges were removed with fine forceps. Hippocampal tissues were sheared and further digested with trypsin, after which the above tissues were filtered to obtain cell suspensions and seeded in six‐well plates coated with poly‐lysine. These neurons were cultured in DMEM/F12 medium supplemented with 10% FBS and 1% penicillin/streptomycin. After the neurons were attached, the medium was replaced with maintenance medium for long‐term culture, with maintenance medium changes occurring every 3 days. To collect medium from primary neurons for treating primary astrocytes, supernatants were harvested from days 14 through 21 of neuronal culture.

Primary glial cells were isolated and cultured from the cerebral cortex of neonatal C57BL/6J mice between days 0 and 2 of life, following a protocol similar to that used for primary neurons. After digestion and filtration, the cells were seeded in six‐well plates, similar to the procedure for primary neurons. Subsequent medium changes were performed at 3‐day intervals. On day 14, microglia and astrocytes were separated via shaking incubation at 180 rpm for 3 h. In the experiment, treating primary astrocytes with recombinant CTSB, as done in previous studies [63], recombinant CTSB was dissolved in basal medium and diluted to a final concentration of 100 nM for 48 h.

### 4.4 Western Blotting

The experimental procedure followed previous studies [64]. Antibody information for Western blotting is shown in Table S4. Protein bands were scanned by the Odyssey Imaging System (LI‐COR, Lincoln, NE, USA).

#### 4.4.1 qRT‐PCR

Serum miRNA was extracted using the Serum miRNA Extraction Kit (Tiangen, Beijing, China). qPCR was carried out on the ABI StepOne Plus system with SYBR Green Premix Ex Taq (Takara, Tokyo, Japan). Relative expression levels were analyzed using the 2^−ΔΔCT^ method, with GAPDH and U6 as internal controls for mRNA and miRNA, respectively. All experiments included biological and technical replicates. Primer sequences are listed in Table S5.

### 4.5 Enzyme‐Linked Immunosorbent Assay

The ELISA was employed to quantify the levels of CTSB, Aβ42, and Aβ40. Serum, hippocampal tissue, and cell supernatant samples were processed and analyzed following the instructions accompanying the kit.

### 4.6 Immunofluorescence Staining

Brain slices were initially washed with PBS and subsequently permeabilized with 0.1% Triton X‐100. After blocking the brain slices with 3% BSA, they were incubated with the corresponding primary antibody overnight. The following day, the slices were treated with a fluorescent secondary antibody at room temperature, protected from light. Fluorescence images were captured using a confocal laser scanning microscope (LSM800, Carl Zeiss) and analyzed using Fiji software.

### 4.7 Three‐Dimensional Morphological Construction and Astrocyte Analysis

Fluorescence images were layer‐scanned at a thickness of 1 µm using a Zeiss laser confocal microscope, and the images were processed and reconstructed in three dimensions using Imaris 9 software. The complexity of the astrocyte arbor and dendritic tree was assessed using Sholl analysis. Additionally, dendritic spines were quantified in two segments on primary branches, specifically 100 and 200 µm from the main body, as described previously [65].

### 4.8 Viruses, Agomirs/Antagomirs, and Stereotaxic Injection

The CTSB overexpression adeno‐associated virus (rAAV2/9) was packaged by OBiO Technology (Shanghai, China). The miR‐96‐5p agomirs, antagomirs, and scrambles were obtained from RiboBio (Guangzhou, China). miR‐96‐5p agomirs and antagomirs were administered as we previously described [60]. After mice were anesthetized and the skin of the head was clipped, two holes were created in the skull and rAAV 1.5 µL (10^13^ TU/mL) or agomirs/antagomirs 2 µL (100 µM) was injected in the DG area (anterior/posterior, −1.9 mm; medial/lateral, ±1.2 mm; dorsal/ventral, −2.2 mm) at 0.2 µL/min. The needle was left in place for 10 min before being slowly withdrawn, sterilized, and sutured to close the mouse wound.

### 4.9 Luciferase Activity Assay

psiCHECK2 containing the WT or CTSB mutation (Mut) 3' untranslated region (3'UTR) was co‐transfected into HEK293T cells with miR‐96‐5p agomirs or NC agomirs. For promoter luciferase reporter assays, pGL3 containing the miR‐96 promoter sequence was co‐transfected into HEK293T cells with pRL‐TK and NeuroD2 or Smad4. Cells were cultured for 48 h and then assayed for luciferase activity according to the manufacturer's instructions.

### 4.10 Behavioral Assays

#### 4.10.1 Open Filed Test

The experiment was conducted in a metal open field, where mice freely explored for 300 s. Their movement and speed were recorded using a digital tracking system. The field was divided into center, side, and corner zones for analysis.

### 4.11 Morris Water Maze

This experiment was conducted as previously described [60]. Mice were trained in a water‐filled circular pool with a submerged hidden platform, using a digital tracking system to monitor their movements. Training lasted six days with three trials per day. On day 8, spatial memory was assessed over a 90 s trial without the platform. Metrics included latency to the original platform location, number of crossings over that location, swimming speed, and time spent in each quadrant.

### 4.12 Barnes Maze

The Barnes maze was used on a circular platform with 18 evenly spaced holes, one of which led to a hidden target box. A digital tracking system recorded mouse movements. Mice were trained over 5 days with two trials per day. On day 7, the target box was removed, and the number of hole explorations within 240 s was recorded.

### 4.13 Statistical Analysis

All data were presented as mean ± SEM and were analyzed using GraphPad Prism software (version 9). To assess the variance between the two groups, a two‐tailed Student's *t*‐test was employed. For determining differences between multiple groups, a one‐way or two‐way analysis of variance (ANOVA) was used, followed by post hoc tests. *p*‐values < 0.05 were considered statistically significant.

## Author Contributions

Ling‐Qiang Zhu conceived and designed the study. Kai Zheng and Jiajie Chen performed the molecular biology experiments and animal experiments. He‐Zhou Huang analyzed the data. Kai Zheng, Jiajie Chen, and Dan Liu collected participant information and serum samples. Kai Zheng, Jiajie Chen, and He‐Zhou Huang wrote the manuscript. Nadezhda Brazhe revised the manuscript. All the authors read and approved the final manuscript.

## Ethics Statement

This study adhered to the Declaration of Helsinki II and was approved by the Ethics Committee of Tongji Hospital, Tongji Medical College, Huazhong University of Science and Technology (TJ‐IRB20210116), and written informed consent was obtained from all participants. Animal studies received approval from the Animal Care and Use Committee of Tongji Medical College (Wuhan, China) with the approval number 3037.

## Conflicts of Interest

K. Z. and L. ‐Q. Z. are authors on patent applications: “Application of miR‐96‐5p combined with CTSB in the diagnosis, screening and treatment of Alzheimer's disease” submitted to the Patent Office of the People's Republic of China (application no. CN202410825627.4). The remaining authors declare no conflicts of interest.

## Supporting information




**Figure S1: Aberrant expression of CTSB in the hippocampus of 3×Tg mice is regulated by miR‐96‐5p**. (A, B) Immunoblot analysis (A) and quantitative analysis (B) of CTSB and GAPDH in the prefrontal cortex of 3‐month‐old and 6‐month‐old WT and 3×Tg mice. N = 6. (C) Statistical analysis of CTSB in different subregions of the hippocampus in 6‐month‐old WT mice and 3×Tg mice. (D) qPCR for relative mRNA expression of *Ctsb* in the hippocampus of 3‐month‐old and 6‐month‐old WT and 3×Tg mice. N = 6. (E) qPCR for miR‐96‐5p of 3‐month‐old WT mice and 3×Tg mice. N = 6. (F) Sequence analysis of the miR‐96‐5p binding region in mammalian *Ctsb* 3'UTR. The mutated sequences in the *Ctsb* 3'UTR used for luciferase assays are listed below. (G) WT or mutated (Mut) 3'UTR of CTSB in the psiCHECK‐2 vector was co‐transfected into HEK293T cells with miR‐96‐5p agomirs (Ago‐miR‐96) or a scramble control (Ago‐NC). Luciferase activity was assessed 48 hours post‐transfection. N = 5. (H, I) qPCR for the relative expression of miR‐96‐5p (H) and *Ctsb* mRNA (I) in N2a cells transfected with Ago‐miR‐96 or Ago‐NC. N = 4. (J, K) qPCR for relative expression of miR‐96‐5p (J) and *Ctsb* mRNA (K) in N2a cells transfected with miR‐96‐5p antagomirs (Anta‐miR‐96) or a scramble control (Anta‐NC). N = 4. (L) GO enrichment analysis of potential miR‐96‐5p target genes. BP: biological process, CC: cellular component, MF: molecular function. *p<0.05, **p<0.01.
**Figure S2: NeuroD2 may be the upstream transcription factor regulating the miR‐96‐5p signaling pathway in AD**. (A, B) qPCR for relative expression of hippocampal pri‐miR‐96 (A) and pre‐miR‐96 (B) in 6‐month‐old WT and 3×Tg mice. N = 6. (C) Relative positional distribution and specific sequences of miR‐96, miR‐182, and miR‐183. (D, E) qPCR for relative expression of pri‐miR‐182 (D) and pri‐miR‐183 (E) in the hippocampus of 6‐month‐old WT and 3×Tg mice. N = 3. (F) Venn diagram illustrating the predicted transcription factors that target miR‐96. (G) qPCR for the six candidate transcription factors with the highest likelihood of targeting miR‐96 in the hippocampus of WT and 3×Tg mice. N = 3. (H) Upper panel: Diagram of the luciferase reporter vector for the pri‐miR‐96 promoter. Lower panel: Analysis of luciferase activity in HEK293T cells following the transfection of luciferase reporter plasmids with pcDNA‐Smad4 or pcDNA‐NeuroD2. N = 3. (I) ChIP‐seq peaks showed binding site of NeuroD2 in promoter of pri‐miR‐96. (J) Immunoblot analysis and quantification of NeuroD2 and GAPDH in the hippocampus of 6‐month‐old WT and 3×Tg mice. N = 6. *p<0.05, **p<0.01.
**Figure S3: Changes of miR‐96‐5p/CTSB signaling pathway in AD serum**. (A) qPCR for the relative expression of miR‐96‐5p in the serum of 6‐month‐old WT and 3×Tg mice. N = 8. (B) ELISA for CTSB levels in the serum of 6‐month‐old WT and 3×Tg mice. N = 8. (C) Correlation analysis between serum miR‐96‐5p and CTSB levels in mice. (D) ROC curve analysis for AD by serum miR‐96‐5p, CTSB, and the combination of miR‐96‐5p and CTSB from healthy controls (HC) (N = 48) and AD patients (N = 52). (E‐L) Participants were categorized based on various characteristics: gender (E), education level (F), smoking status (G), drinking (H), hypertension (I), coronary heart disease (CHD) (J), diabetes (K), and hyperlipidemia (L). qPCR was performed to measure the relative expression levels of miR‐96‐5p in serum. *p<0.05, **p<0.01.
**Figure S4: Aberrant miR‐96‐5p signaling may be associated with carrying *APOE ε4***. (A) qPCR for the relative expression of miR‐96‐5p in the serum of *APOE3/3* and *APOE4/4* mice. N = 6. (B, C) Representative immunofluorescence staining images (B) and fluorescence intensity statistics (C) for miR‐96‐5p in the DG region of *APOE3/3* and *APOE4/4* mice. N = 10 from 3 to 4 mice. (D) Immunoblot analysis and quantification of NeuroD2 and GAPDH in the hippocampus of *APOE3/3* and *APOE4/4* mice. N = 3. *p<0.05, **p<0.01.
**Figure S5: Characterization of cellular distribution of miR‐96‐5p/CTSB signaling pathway in AD hippocampus**. (A‐C) Representative immunofluorescence staining images and fluorescence intensity statistics (C) for CTSB in the hippocampal CA1 region (A) and CA3 region (B) of 6‐month‐old WT and 3×Tg mice. N = 10 from 3 to 4 mice. (D‐F) Double immunofluorescence staining for the expression of CTSB (green) in microglia (red) (D), astrocytes (red) (E), and oligodendrocytes (red) (F) in the DG region of 6‐month‐old WT and 3×Tg mice. (G‐I) Double immunofluorescence staining for the expression of miR‐96‐5p (red) in microglia (green) (G), astrocytes (green) (H), and oligodendrocytes (green) (I) in the DG region of 6‐month‐old WT and 3×Tg mice. *p<0.05, **p<0.01.
**Figure S6: Abnormal miR‐96‐5p/CTSB signaling in AD may be associated with Aβ pathology**. (A) Fluorescence intensity statistics for miR‐96 and CTSB in the DG region of WT and APP/PS1 (A/P) mice. N = 10 from 3 mice. (B) Immunoblot analysis and quantification of CTSB and GAPDH in the hippocampus of WT and A/P mice. N = 6. (C, D) qPCR for relative expression of hippocampal *Ctsb* mRNA (C) and miR‐96‐5p (D) in WT and A/P mice. N = 6. (E) Fluorescence intensity statistics for miR‐96 and CTSB in the DG region of WT and P301L mice. N = 10 from 3 mice. (F) Immunoblot analysis and quantitative assessment of hippocampal CTSB and GAPDH in WT and P301L mice. N = 6. (G, H) qPCR for relative expression of hippocampal *Ctsb* mRNA (G) and miR‐96‐5p (H) in WT and P301L mice. N = 6. (I) ELISA for the concentrations of Aβ42 and Aβ40 in the hippocampus of WT and 3×Tg mice, and their ratios were calculated. N = 12. *p<0.05, **p<0.01.
**Figure S7: Manipulation of hippocampal miR‐96‐5p/CTSB signaling pathway in WT mice and activation of astrocytes by conditioned medium of primary neurons in APP/PS1 mice**. (A) Immunoblot analysis and quantification of CTSB and GAPDH following the injection of miR‐96‐5p antagomirs (Anta‐miR‐96) or a scramble control (An‐NC) in the DG region of 6‐month‐old WT mice. N = 6. (B, C) qPCR for the relative expression of miR‐96‐5p (B) and *Ctsb* mRNA(C) in the hippocampus of 6‐month‐old WT mice following the injection of Anta‐miR‐96 or a scrambled control (An‐NC). N = 6. (D) Schematic representation of the DG region of 5‐month‐old WT mice injected with rAAV2/9‐hSyn1‐CTSB‐P2A‐EGFP or a control virus. (E) Immunoblot analysis and quantitative assessment of CTSB and GAPDH following the injection of rAAV2/9‐hSyn1‐CTSB‐P2A‐EGFP (CTSB OE) or a control virus (Ctrl) into the DG region of 5‐month‐old WT mice. (F) Representative immunofluorescence staining images of primary neuronal morphology after overexpression of CTSB. (G) Cell viability of primary neurons after overexpression of CTSB detected by CCK8 assay. N = 5. (H) qPCR for the relative expression of *Megf10* and *Mertk* mRNA in primary astrocytes. N = 6. (I) qPCR for the relative expression of *IL‐1β*, *IL‐6*, and tumor necrosis *TNF‐α* in primary astrocytes. N = 6. (J, K) Representative immunofluorescence staining (J) and statistics (K) of primary microglia. N = 15. (L) Immunoblot analysis and quantitative assessment of CTSB and GAPDH in primary neurons derived from WT or APP/PS1 mice. N = 6. (M) ELISA for relative expression of CTSB in the conditioned medium of primary neurons derived from WT or APP/PS1 mice. N = 8. (N‐P) Immunofluorescence staining of primary astrocytes (N) along with cell size (O) and fluorescence intensity (P) statistics of GFAP‐positive astrocytes. N = 20. (Q) qPCR for RNA levels of *IL‐1β*, *IL‐6*, and *TNF‐α* in primary astrocytes. N = 6. *p<0.05, **p<0.01.
**Figure S8: Restoration of miR‐96‐5p signaling pathway does not affect the emotional state of 3×Tg mice**. (A, B) Statistics of residence time (A) and movement speed (B) of mice in each region during open field experiments following the injection of miR‐96‐5p agomirs (A‐miR‐96) or a scrambled control (A‐NC) in the DG region of 6‐month‐old WT and 3×Tg mice. N = 8.

**Supporting Table 1**: mco270368‐sup‐0001‐tableS1.xlsx

**Supporting Table 2**: mco270368‐sup‐0001‐tableS2.xlsx

**Supporting Table 3**: mco270368‐sup‐0001‐tableS3.xlsx

**Supporting Table 4**: mco270368‐sup‐0001‐tableS4.xlsx

**Supporting Table 5**: mco270368‐sup‐0001‐tableS5.xlsx

**Supporting Table 6**: mco270368‐sup‐0001‐tableS6.xlsx

## Data Availability

All data associated with this study are present in the paper or the Supporting Information.
