# Supplementary material for: Targeting the miR‐96‐5p/Cathepsin B Pathway to Alleviate Neuron‐Derived Neuroinflammation in Alzheimer's Disease
Source: MedComm (2020). 2025 Sep 6;6(9):e70368. doi: 10.1002/mco2.70368 (PMC12413563; doi:10.1002/mco2.70368)
Supplement: Supplementary file 1 — Figure S1: Aberrant expression of CTSB in the hippocampus of 3×Tg mice is regulated by miR‐96‐5p. (A, B) Immunoblot analysis (A) and quantitative analysis (B) of CTSB and GAPDH in the prefrontal cortex of 3‐month‐old and 6‐month‐old WT and 3×Tg mice. N = 6. (C) Statistical analysis of CTSB in different subregions of the hippocampus in 6‐month‐old WT mice and 3×Tg mice. (D) qPCR for relative mRNA expression of Ctsb in the hippocampus of 3‐month‐old and 6‐month‐old WT and 3×Tg mice. N = 6. (E) qPCR for miR‐96‐5p of 3‐month‐old WT mice and 3×Tg mice. N = 6. (F) Sequence analysis of the miR‐96‐5p binding region in mammalian Ctsb 3'UTR. The mutated sequences in the Ctsb 3'UTR used for luciferase assays are listed below. (G) WT or mutated (Mut) 3'UTR of CTSB in the psiCHECK‐2 vector was co‐transfected into HEK293T cells with miR‐96‐5p agomirs (Ago‐miR‐96) or a scramble control (Ago‐NC). Luciferase activity was assessed 48 hours post‐transfection. N = 5. (H, I) qPCR for the relative expression of miR‐96‐5p (H) and Ctsb mRNA (I) in N2a cells transfected with Ago‐miR‐96 or Ago‐NC. N = 4. (J, K) qPCR for relative expression of miR‐96‐5p (J) and Ctsb mRNA (K) in N2a cells transfected with miR‐96‐5p antagomirs (Anta‐miR‐96) or a scramble control (Anta‐NC). N = 4. (L) GO enrichment analysis of potential miR‐96‐5p target genes. BP: biological process, CC: cellular component, MF: molecular function. *p<0.05, **p<0.01. Figure S2: NeuroD2 may be the upstream transcription factor regulating the miR‐96‐5p signaling pathway in AD. (A, B) qPCR for relative expression of hippocampal pri‐miR‐96 (A) and pre‐miR‐96 (B) in 6‐month‐old WT and 3×Tg mice. N = 6. (C) Relative positional distribution and specific sequences of miR‐96, miR‐182, and miR‐183. (D, E) qPCR for relative expression of pri‐miR‐182 (D) and pri‐miR‐183 (E) in the hippocampus of 6‐month‐old WT and 3×Tg mice. N = 3. (F) Venn diagram illustrating the predicted transcription factors that target miR‐96. (G) qPCR for the six cand [file MCO2-6-e70368-s001.docx]

**Targeting the miR-96-5p/Cathepsin B Pathway to Alleviate Neuron-Derived Neuroinflammation in Alzheimer's Disease**

Kai Zheng^1^, He-Zhou Huang^2^, Dan Liu^2^, Nadezhda Brazhe^3^, Jiajie Chen^1*^, Ling-Qiang Zhu^2*^

^1^Department of Geriatrics, Tongji Hospital, Tongji Medical College, Huazhong University of Science and Technology, Wuhan, China.

^2^Department of Pathophysiology, School of Basic Medicine, Tongji Medical College, Huazhong University of Science and Technology, Wuhan, Hubei 430030, China

^3^Department of Biophysics, Faculty of biology, Moscow State University, Russia

^*^Corresponding author. Email: zhulq@mail.hust.edu.cn (L.-Q.Z.); chenjiajie@tjh.tjmu.edu.cn (J.C.)


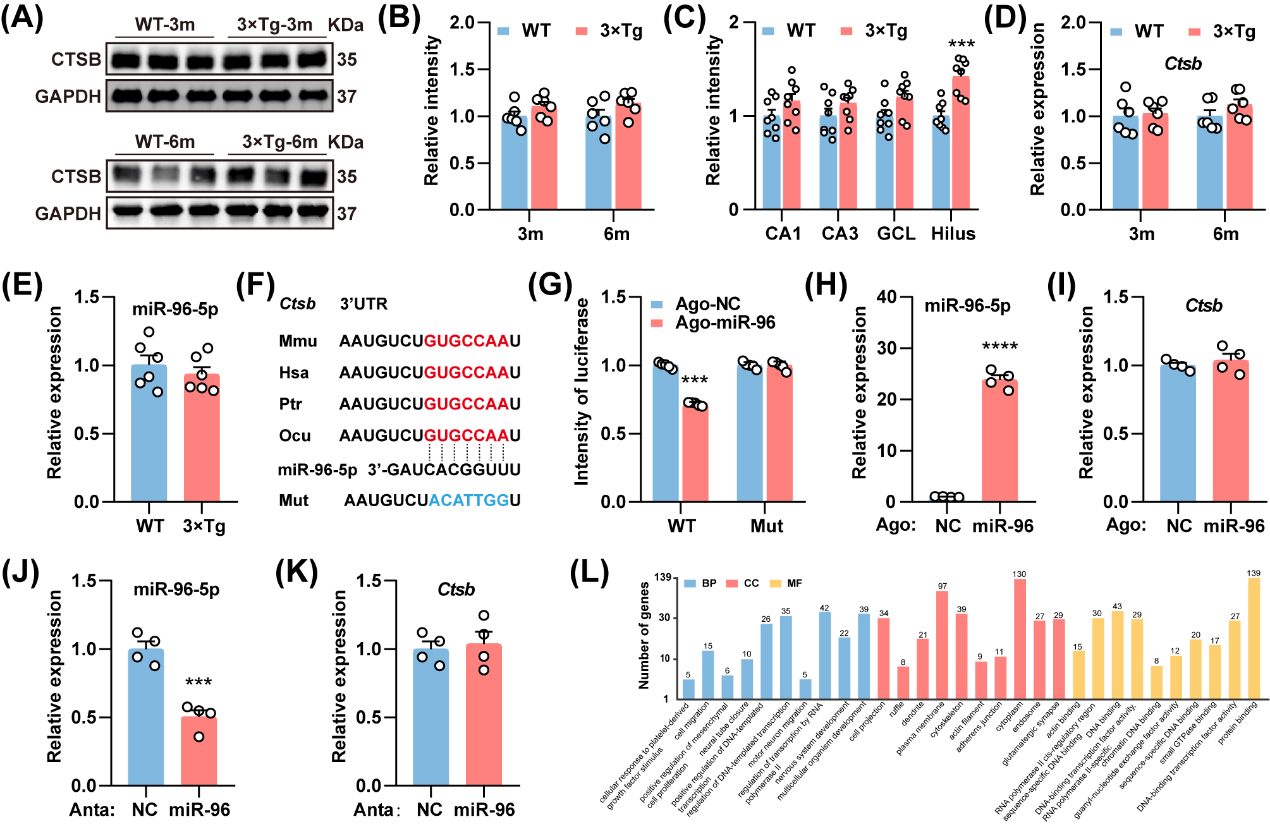


**Figure S1. Aberrant expression of CTSB in the hippocampus of 3×Tg mice is regulated by miR-96-5p.**

(A, B) Immunoblot analysis (A) and quantitative analysis (B) of CTSB and GAPDH in the prefrontal cortex of 3-month-old and 6-month-old WT and 3×Tg mice. N = 6. (C) Statistical analysis of CTSB in different subregions of the hippocampus in 6-month-old WT mice and 3×Tg mice. (D) qPCR for relative mRNA expression of *Ctsb* in the hippocampus of 3-month-old and 6-month-old WT and 3×Tg mice. N = 6. (E) qPCR for miR-96-5p of 3-month-old WT mice and 3×Tg mice. N = 6. (F) Sequence analysis of the miR-96-5p binding region in mammalian *Ctsb* 3'UTR. The mutated sequences in the *Ctsb* 3'UTR used for luciferase assays are listed below. (G) WT or mutated (Mut) 3'UTR of CTSB in the psiCHECK-2 vector was co-transfected into HEK293T cells with miR-96-5p agomirs (Ago-miR-96) or a scramble control (Ago-NC). Luciferase activity was assessed 48 hours post-transfection. N = 5. (H, I) qPCR for the relative expression of miR-96-5p (H) and *Ctsb* mRNA (I) in N2a cells transfected with Ago-miR-96 or Ago-NC. N = 4. (J, K) qPCR for relative expression of miR-96-5p (J) and *Ctsb* mRNA (K) in N2a cells transfected with miR-96-5p antagomirs (Anta-miR-96) or a scramble control (Anta-NC). N = 4. (L) GO enrichment analysis of potential miR-96-5p target genes. BP: biological process, CC: cellular component, MF: molecular function. **P*＜0.05, ***P*＜0.01.


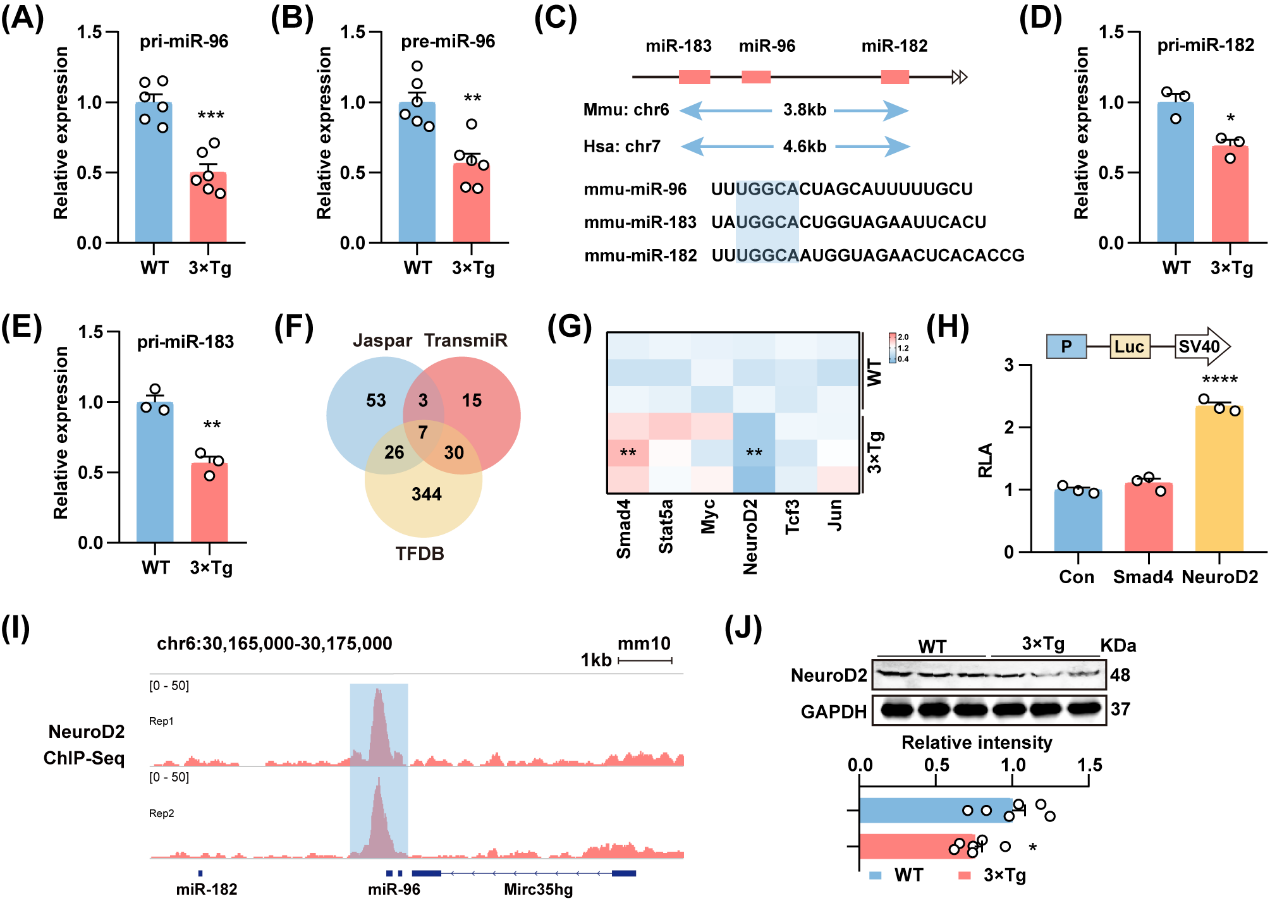


**Figure S2. NeuroD2 may be the upstream transcription factor regulating the miR-96-5p signaling pathway in AD.**

(A, B) qPCR for relative expression of hippocampal pri-miR-96 (A) and pre-miR-96 (B) in 6-month-old WT and 3×Tg mice. N = 6. (C) Relative positional distribution and specific sequences of miR-96, miR-182, and miR-183. (D, E) qPCR for relative expression of pri-miR-182 (D) and pri-miR-183 (E) in the hippocampus of 6-month-old WT and 3×Tg mice. N = 3. (F) Venn diagram illustrating the predicted transcription factors that target miR-96. (G) qPCR for the six candidate transcription factors with the highest likelihood of targeting miR-96 in the hippocampus of WT and 3×Tg mice. N = 3. (H) Upper panel: Diagram of the luciferase reporter vector for the pri-miR-96 promoter. Lower panel: Analysis of luciferase activity in HEK293T cells following the transfection of luciferase reporter plasmids with pcDNA-Smad4 or pcDNA-NeuroD2. N = 3. (I) ChIP-seq peaks showed binding site of NeuroD2 in promoter of pri-miR-96. (J) Immunoblot analysis and quantification of NeuroD2 and GAPDH in the hippocampus of 6-month-old WT and 3×Tg mice. N = 6. **P*＜0.05, ***P*＜0.01.


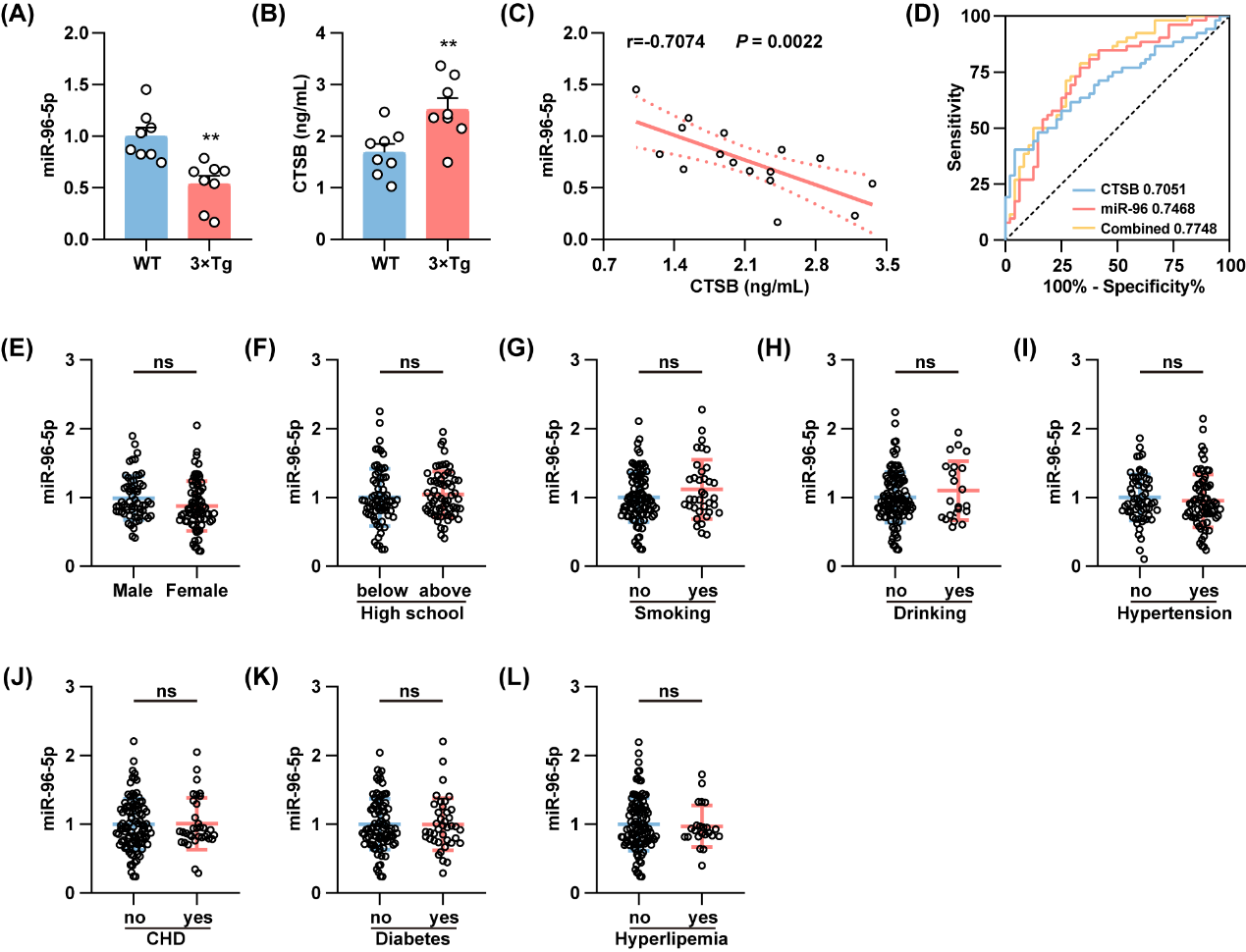


**Figure S3. Changes of miR-96-5p/CTSB signaling pathway in AD serum.**

(A) qPCR for the relative expression of miR-96-5p in the serum of 6-month-old WT and 3×Tg mice. N = 8. (B) ELISA for CTSB levels in the serum of 6-month-old WT and 3×Tg mice. N = 8. (C) Correlation analysis between serum miR-96-5p and CTSB levels in mice. (D) ROC curve analysis for AD by serum miR-96-5p, CTSB, and the combination of miR-96-5p and CTSB from healthy controls (HC) (N = 48) and AD patients (N = 52). (E-L) Participants were categorized based on various characteristics: gender (E), education level (F), smoking status (G), drinking (H), hypertension (I), coronary heart disease (CHD) (J), diabetes (K), and hyperlipidemia (L). qPCR was performed to measure the relative expression levels of miR-96-5p in serum. **P*＜0.05, ***P*＜0.01.


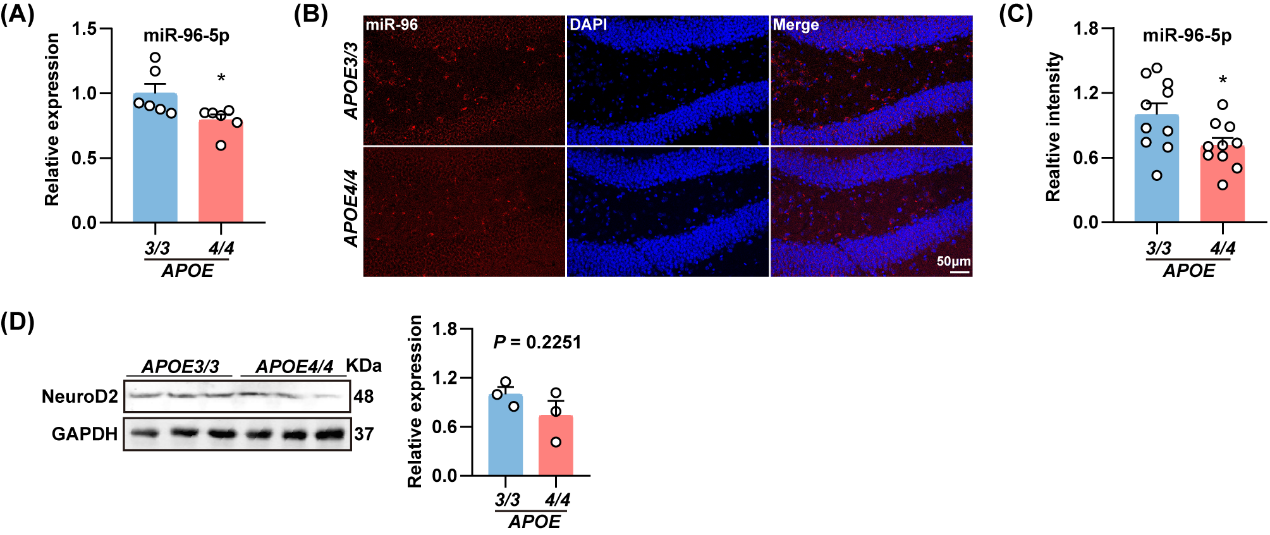


**Figure S4. Aberrant miR-96-5p signaling may be associated with carrying *APOE ε4.***

(A) qPCR for the relative expression of miR-96-5p in the serum of *APOE3/3* and *APOE4/4* mice. N = 6. (B, C) Representative immunofluorescence staining images (B) and fluorescence intensity statistics (C) for miR-96-5p in the DG region of *APOE3/3* and *APOE4/4* mice. N = 10 from 3 to 4 mice. (D) Immunoblot analysis and quantification of NeuroD2 and GAPDH in the hippocampus of *APOE3/3* and *APOE4/4* mice. N = 3. **P*＜0.05, ***P*＜0.01.


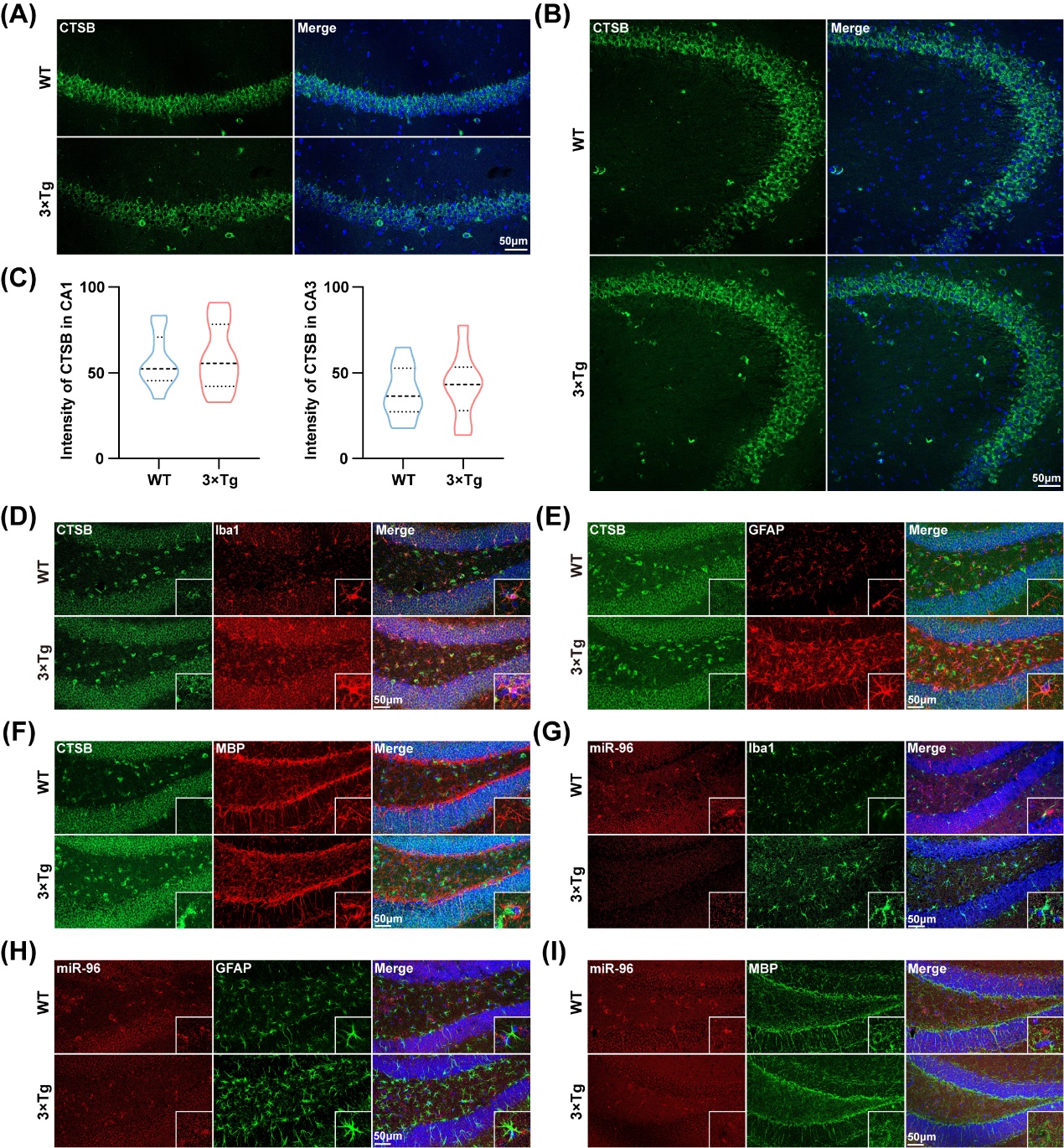


**Figure S5. Characterization of cellular distribution of miR-96-5p/CTSB signaling pathway in AD hippocampus.**

(A-C) Representative immunofluorescence staining images and fluorescence intensity statistics (C) for CTSB in the hippocampal CA1 region (A) and CA3 region (B) of 6-month-old WT and 3×Tg mice. N = 10 from 3 to 4 mice. (D-F) Double immunofluorescence staining for the expression of CTSB (green) in microglia (red) (D), astrocytes (red) (E), and oligodendrocytes (red) (F) in the DG region of 6-month-old WT and 3×Tg mice. (G-I) Double immunofluorescence staining for the expression of miR-96-5p (red) in microglia (green) (G), astrocytes (green) (H), and oligodendrocytes (green) (I) in the DG region of 6-month-old WT and 3×Tg mice. **P*＜0.05, ***P*＜0.01.


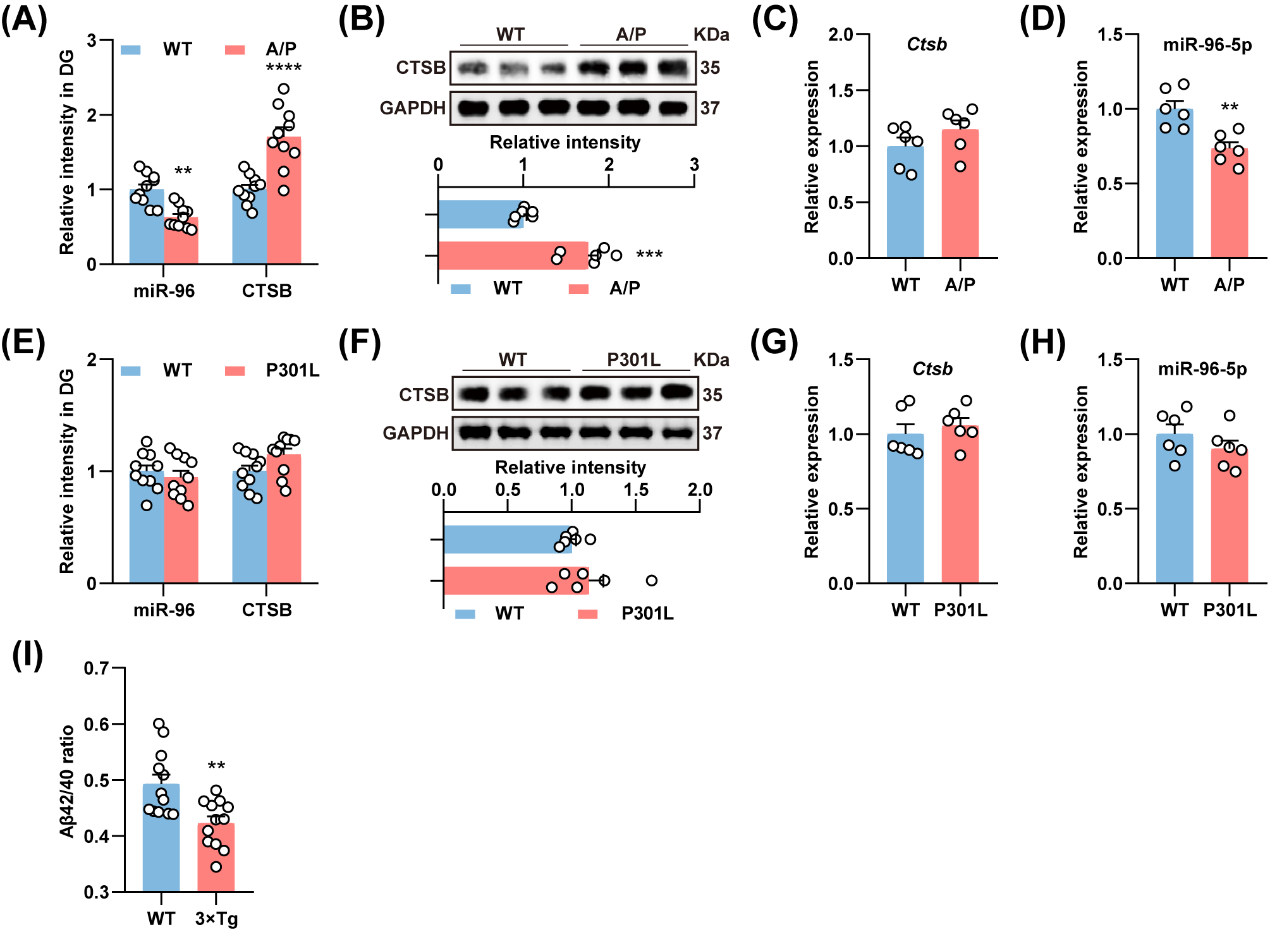


**Figure S6. Abnormal miR-96-5p/CTSB signaling in AD may be associated with Aβ pathology.**

(A) Fluorescence intensity statistics for miR-96 and CTSB in the DG region of WT and APP/PS1 (A/P) mice. N = 10 from 3 mice. (B) Immunoblot analysis and quantification of CTSB and GAPDH in the hippocampus of WT and A/P mice. N = 6. (C, D) qPCR for relative expression of hippocampal *Ctsb* mRNA (C) and miR-96-5p (D) in WT and A/P mice. N = 6. (E) Fluorescence intensity statistics for miR-96 and CTSB in the DG region of WT and P301L mice. N = 10 from 3 mice. (F) Immunoblot analysis and quantitative assessment of hippocampal CTSB and GAPDH in WT and P301L mice. N = 6. (G, H) qPCR for relative expression of hippocampal *Ctsb* mRNA (G) and miR-96-5p (H) in WT and P301L mice. N = 6. (I) ELISA for the concentrations of Aβ42 and Aβ40 in the hippocampus of WT and 3×Tg mice, and their ratios were calculated. N = 12. **P*＜0.05, ***P*＜0.01.


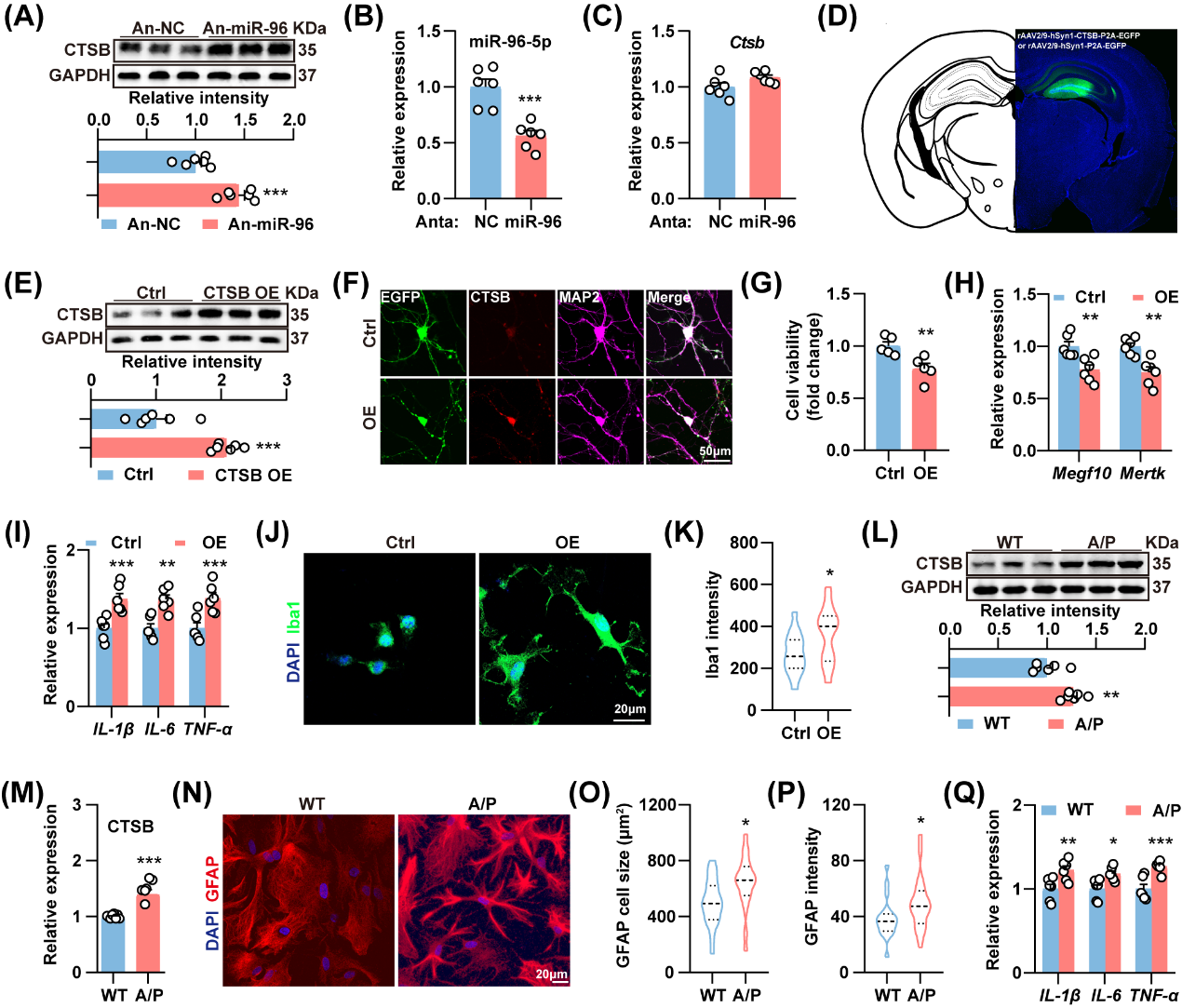


**Figure S7. Manipulation of hippocampal miR-96-5p/CTSB signaling pathway in WT mice and activation of astrocytes by conditioned medium of primary neurons in APP/PS1 mice.**

(A) Immunoblot analysis and quantification of CTSB and GAPDH following the injection of miR-96-5p antagomirs (Anta-miR-96) or a scramble control (An-NC) in the DG region of 6-month-old WT mice. N = 6. (B, C) qPCR for the relative expression of miR-96-5p (B) and *Ctsb* mRNA(C) in the hippocampus of 6-month-old WT mice following the injection of Anta-miR-96 or a scrambled control (An-NC). N = 6. (D) Schematic representation of the DG region of 5-month-old WT mice injected with rAAV2/9-hSyn1-CTSB-P2A-EGFP or a control virus. (E) Immunoblot analysis and quantitative assessment of CTSB and GAPDH following the injection of rAAV2/9-hSyn1-CTSB-P2A-EGFP (CTSB OE) or a control virus (Ctrl) into the DG region of 5-month-old WT mice. (F) Representative immunofluorescence staining images of primary neuronal morphology after overexpression of CTSB. (G) Cell viability of primary neurons after overexpression of CTSB detected by CCK8 assay. N = 5. (H) qPCR for the relative expression of *Megf10* and *Mertk* mRNA in primary astrocytes. N = 6. (I) qPCR for the relative expression of *IL-1β*, *IL-6*, and tumor necrosis *TNF-α* in primary astrocytes. N = 6. (J, K) Representative immunofluorescence staining (J) and statistics (K) of primary microglia. N = 15. (L) Immunoblot analysis and quantitative assessment of CTSB and GAPDH in primary neurons derived from WT or APP/PS1 mice. N = 6. (M) ELISA for relative expression of CTSB in the conditioned medium of primary neurons derived from WT or APP/PS1 mice. N = 8. (N-P) Immunofluorescence staining of primary astrocytes (N) along with cell size (O) and fluorescence intensity (P) statistics of GFAP-positive astrocytes. N = 20. (Q) qPCR for RNA levels of *IL-1β*, *IL-6*, and *TNF-α* in primary astrocytes. N = 6. **P*＜0.05, ***P*＜0.01.


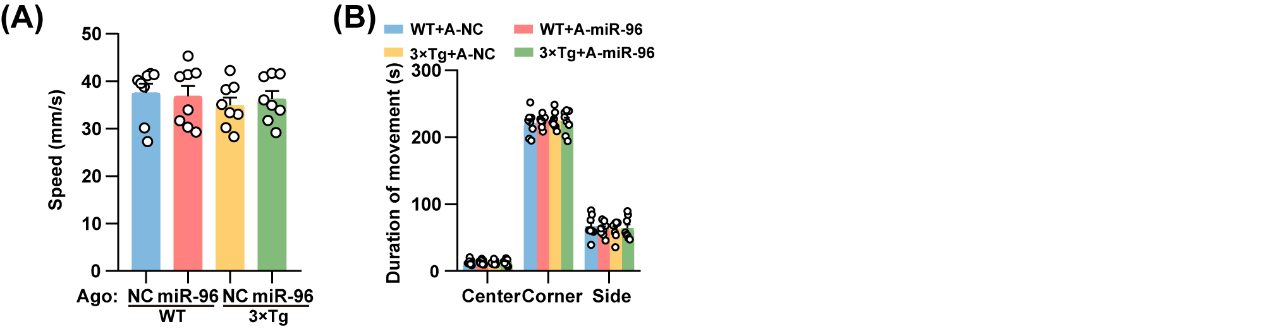


**Figure S8. Restoration of miR-96-5p signaling pathway does not affect the emotional state of 3×Tg mice.**

(A, B) Statistics of residence time (A) and movement speed (B) of mice in each region during open field experiments following the injection of miR-96-5p agomirs (A-miR-96) or a scrambled control (A-NC) in the DG region of 6-month-old WT and 3×Tg mice. N = 8.
